# Interventions aimed at reducing problems in adult patients discharged from hospital to home: a systematic meta-review

**DOI:** 10.1186/1472-6963-7-47

**Published:** 2007-04-04

**Authors:** Patriek Mistiaen, Anneke L Francke, Else Poot

**Affiliations:** 1 NIVEL, Netherlands Institute for Health Services Research, P.O. Box 1568, 3500 BN Utrecht, the Netherlands; 2 The Netherlands Centre of Excellence in Nursing (LEVV), P.O. Box 3135, 3502 GC Utrecht, the Netherlands

## Abstract

**Background:**

Many patients encounter a variety of problems after discharge from hospital and many discharge (planning and support) interventions have been developed and studied. These primary studies have already been synthesized in several literature reviews with conflicting conclusions. We therefore set out a systematic review of the reviews examining discharge interventions. The objective was to synthesize the evidence presented in literature on the effectiveness of interventions aimed to reduce post-discharge problems in adults discharged home from an acute general care hospital.

**Methods:**

A comprehensive search of seventeen literature databases and twenty-five websites was performed for the period 1994–2004 to find relevant reviews. A three-stage inclusion process consisting of initial sifting, checking full-text papers on inclusion criteria, and methodological assessment, was performed independently by two reviewers. Data on effects were synthesized by use of narrative and tabular methods.

**Results:**

Fifteen systematic reviews met our inclusion criteria. All reviews had to deal with considerable heterogeneity in interventions, populations and outcomes, making synthesizing and pooling difficult.

Although a statistical significant effect was occasionally found, most review authors reached no firm conclusions that the discharge interventions they studied were effective.

We found limited evidence that some interventions may improve knowledge of patients, may help in keeping patients at home or may reduce readmissions to hospital. Interventions that combine discharge planning and discharge support tend to lead to the greatest effects. There is little evidence that discharge interventions have an impact on length of stay, discharge destination or dependency at discharge. We found no evidence that discharge interventions have a positive impact on the physical status of patients after discharge, on health care use after discharge, or on costs.

**Conclusion:**

Based on fifteen high quality systematic reviews, there is some evidence that some interventions may have a positive impact, particularly those with educational components and those that combine pre-discharge and post-discharge interventions. However, on the whole there is only limited summarized evidence that discharge planning and discharge support interventions have a positive impact on patient status at hospital discharge, on patient functioning after discharge, on health care use after discharge, or on costs.

## Background

Going back home from hospital is not always a smooth process. Many studies from all over the world have repeatedly reported that many people who have been discharged from hospital to home, especially the elderly, encounter a variety of problems in the first weeks after their return home. Problems after discharge include dependence on others with regard to household activities [1-6], lower levels of independence in activities of daily living and self-care deficits [2,3,5-12], difficulty with reading medication labels or instilling eyedrops [13,14], not getting the help they needed [4,5,13,15-23], not being aware of available services [24-26], informational needs [4,13,26-30], symptom distress [28,31-33], social problems [34] and emotional problems as anxiety and uncertainty [7,29,35]. The post-discharge problems seem to be more common with increased age and in women [36] and may lead to further complications and unplanned hospital readmissions.

In addition, lengths of hospital stay have dropped steeply in the last few decades, e.g. from 6.5 days in 1985 to 4.8 days in 2003 in the USA (with the greatest decline for people aged 65 years and older [37]), from 10.5 days in 1985 to 6.9 days in 2003 in the European Union [38], and from 12.5 days in 1985 to 7.3 days in 2003 in the Netherlands [39]. Consequently, the time available to a healthcare team to adequately prepare patients for discharge has virtually evaporated [40].

Discharge planning and aftercare initiatives have received much and increased attention over the past few years as a result. Rorden & Taft defined discharge planning as 'a process made up of several steps or phases whose immediate goal is to anticipate changes in patient care needs and whose long-term goal is to ensure continuity of health care' [41]. We defined discharge interventions as in-hospital interventions or interventions after discharge performed (partly) by hospital-based professionals, explicitly targeted to smooth the transition from hospital to home or to prevent or diminish problems after hospital discharge.

Many studies were performed with various forms of discharge planning and aftercare, e.g. screening patients with a high risk of post discharge problems [42,43], intensive in-hospital discharge preparation [44], discharge rounds [45,46], transitional and intermediate care units [32,47-50], written information leaflets [51], liaison nurses and discharge coordinators [52-55], clinical nurse specialists [56-58], home visits prior to discharge [59,60], preventive home visits of district nurses after discharge [61-63], post-hospital support programs [7,64-68], telephone follow-up after discharge [69-72], discharge planning protocols [18,73], ameliorated communication between hospital and primary care providers [74,75], and many others [76,77].

These 'discharge interventions' mostly aim to smoothen the discharge itself (generally measured by length of stay and discharge destination) or to prevent, ease or solve problems in patient's functioning after discharge (generally measured by function-measures) or to prevent readmissions to the hospital (which are generally seen as a proxy for patient problems after discharge) or to lower health care costs, related to hospital readmissions and treatment of post discharge problems.

Reviews of these studies come to different conclusions on the effectiveness of these interventions, varying from "*Discharge planning and support teams are cost effective and should be in place universally*" [78] to "*The impact of discharge planning on readmission rates, hospital length of stay, health outcomes and cost is uncertain*" [79] to '*In general, the evidence is a mixture of benefit, deficit and uncertainty, due to the complexity and variability of the interventions and methodological problems with the evaluations*' [80] and "*Evidence from RCT's is not available to support the general adoption of discharge planning protocols, geriatric assessment processes or discharge support schemes as means of improving discharge outcomes*" [81].

The mixed results of the reviews may, however, be caused by different study populations, heterogeneity of interventions, or a variety of outcomes that have been chosen. A lot of questions with regard to the optimal content and the organization of discharge planning and support remain unanswered. We therefore set out a systematic review of reviews dealing with discharge interventions.

As mentioned earlier, we defined discharge interventions as in-hospital interventions or interventions after discharge performed (partly) by hospital-based professionals, explicitly targeted to smooth the transition from hospital to home or to prevent or diminish problems after hospital discharge. These can roughly be classified in two groups:

- *Discharge preparation*: interventions that mainly take place during admission in the hospital, with the objective of organizing care and preparing patients in such a way that the length of hospital stay is as short as possible for most patients, that the condition of most patients is such that they can be discharged home and not into institutional care, that they will need as little care as possible post discharge, and that care (organizations) needed after discharge are informed and organized as well as possible, so that patients will not have unmet needs, will not have to be readmitted and will not die due to complications or deterioration after discharge.

- *Discharge support/aftercare*: interventions that mainly take place after discharge from hospital and that are targeted to prevent, ease or solve problems after discharge in order to prevent readmissions to hospital or admissions to institutional care and to maximize recovery and improve functional, emotional, social and health status in the post-discharge period.

Besides this rough two categories classification system, we considered the categorization of discharge interventions put forward by Parker et al. [81] as a useful additional framework for ordering the results of the included reviews. Parker et al. have four broad classes of 'discharge arrangements': comprehensive discharge planning protocols, comprehensive geriatric assessment programmes, discharge support arrangements and educational interventions, all of which can be either generic or disease specific. They define these as follows:

- 'Comprehensive discharge planning protocols' are interventions involving standardised actions or interventions carried out by an individual, including assessment, coordination and implementation of the discharge plan, which project post-discharge needs with the aim of preventing unnecessary readmission, maintaining the health status of patients or lessening carers' burdens.

- 'Comprehensive geriatric assessment (CGA) programmes' are programmes based either in hospital or supporting older people recently discharged from hospital. In CGA programmes the multidisciplinary, multidimensional nature of the assessment of health, rehabilitation and social care needs is formalized, often using standardized assessment instruments. The results of these formal assessments are then used either to inform or prompt treatment and management recommendations, which may be carried out in dedicated inpatient units, provided as recommendations to the referring physician or team, or delivered in the patient's home or other ambulatory care setting such as the day hospital or outpatient clinic. Discharge planning is usually regarded as an important component of inpatient CGA programmes, although most are not focused on discharge itself, but on improving functional health status, and thereby independent living, through medical intervention and rehabilitation.

- 'Discharge support arrangements' are schemes that are designed to provide support for (older) people after experiencing discharge from inpatient hospital care. These are interventions in which hospital or community staff are in contact with the patient around the time of hospital discharge, with the specific intention of providing support during the post-discharge period. The interventions may be limited to a post-discharge telephone contact at one extreme, or, at the other extreme, involve teams of professionals providing services in the patient's home after discharge from hospital.

- 'Educational interventions' are interventions targeted at patients undergoing discharge from hospital that are intended to improve their ability to manage aspects of their care after discharge through the provision of information or more active education. The interventions may be limited to education, or supplemented by other activities such as home visits or telephone calls after discharge.

The objective of this meta-review was to identify, appraise and synthesize the evidence presented in reviews of the literature for the effectiveness of discharge interventions in reducing post-discharge problems in adults discharged home from an acute general care hospital. In addition to problems in patient's functioning after discharge we sought for evidence about the effects of discharge interventions on discharge status and on health care services use and costs after discharge.

The following questions were addressed:

- What are the effects of 'discharge interventions' on the discharge status of patients?

(length of hospital stay, discharge destination, dependency at discharge)

- What are the effects of 'discharge interventions' on the functioning of patients in the first 3 months after discharge?

(physical status, emotional status, social status, health status)

- What are the effects of these interventions on health care services use and costs in the first 3 months after discharge?

(readmissions, use of health care services post discharge, costs)

Outcomes in carers or relatives were not considered.

## Methods

### Data sources

We searched for reviews of the literature and reviews that are part of evidence-based guidelines containing synthesized evidence relating to discharge planning and support interventions aimed at preventing or diminishing problems in adult patients following hospital discharge.

Searches were performed in seventeen literature databases and on twenty-five websites, which are listed in Appendix 1 (see Additional file 1). All databases were searched from 1994 (or from their inception if this was later than 1994) until December 2004.

A search strategy for PUBMED was developed; which was partly based on the search filters of the Dutch Cochrane Centre for searching systematic reviews and for searching guidelines in PUBMED [82]. Suitable search strategies were developed for the other databases, as adaptations of the PUBMED search. No limits were applied where languages were concerned. All detailed search strategies can be found in Appendix 2 (see Additional file 2).

The words "discharge planning", "aftercare", "hospital discharge" and "continuity of care" (or equivalents in Dutch, French or German for the non-English sites) were sequentially entered in the search frame of the sites, for the purpose of searching the websites to find systematic reviews as part of a guideline.

The hits of all searches were entered into Reference Manager^©^, duplicates were sifted out in this program, and the inclusion process were executed thereafter.

### Study selection

The manuscripts had to fulfil all of the following criteria in order to be included:

- The manuscript is a systematic review of the literature, either as an independent manuscript or as a part of a guideline (we considered a review as a systematic review if at least two out of three of the following criteria were met: a search strategy was reported, a search was performed in Pubmed at least, and the included studies were subjected to some kind of methodological assessment)

- The review concerns 'discharge interventions' (= in-hospital interventions or interventions after discharge performed (partly) by hospital-based professionals, explicitly targeted to smooth the transition from hospital to home or to prevent or diminish problems after hospital discharge)

- The interventions discussed in the review relate to adult patients discharged home from an acute general care hospital, who were admitted for a primarily physical problem

- The outcomes studied in the review concern patient status at discharge, patient functioning after discharge, or health care service use and costs after discharge

- The outcomes studied in the review are measured within 3 months after discharge from hospital

- None of the exclusion criteria listed below are met

- The review has sufficient methodological quality (= Overview Quality Assessment Questionnaire score ≥ 5 [83-85])

Publications were excluded when:

- They were primary research studies

- The outcomes in the review were only reported for carers or professionals

- The review involved only paediatric or psychiatric patients

- The review involved only emergency department (ED) patients or one-day stay procedures

- The review concerned interventions that are primarily intended to address the problems of caregivers rather than of patients

- The experimental interventions discussed in the review are performed after discharge solely by primary care providers

Since there is no generally accepted definition of what a postdischarge period means, and the duration of postdischarge problems may vary for different illnesses and treatment procedures, the choice of a time period of 3 months as inclusion criterion had to be arbitrary. There is evidence, however, that most postdischarge problems occur in the period immediately after discharge: Naylor states in her review [86] that *'4 to 6 weeks post discharge represents a critical period when many elders are at highest risk for poor discharge outcomes*' and empirical research in a mixed population has shown that postdischarge problems are greater at 7 days post discharge than at 30 days post discharge [43]. Moreover, three months is a period for which it is reasonable to assume that outcomes can be related to the intervention around or in the first month after discharge.

A three-stage inclusion process was applied. Titles and abstracts of articles identified from the search strategies were screened in the first stage of initial sifting, in order to determine their relevance and whether they fulfilled the inclusion criteria. For each study the criteria were judged from top to bottom of the inclusion criteria referred to; no further analysis was done on the subsequent criteria as soon as one criterion was not met. In this first stage (which is more focused on excluding than on including), one reviewer screened all references and the second reviewer independently checked a 10% random sample of the references. If agreement between the two reviewers on whether to exclude studies was lower than 95% for the 10% sample, the second reviewer would proceed to check the other 90% of the sample. In addition, 10% of the references that were excluded by the first reviewer were checked by a second reviewer. When the title and/or abstract provided insufficient information to determine relevance, full paper copies of the articles were ordered and they proceeded to the second stage. In case articles were published in a language in which the reviewers were not fluent, assistance was sought from other colleagues who mastered that language.

In the second stage, two reviewers independently examined all full paper copies of the articles selected in the first stage, in order to determine whether they fulfilled the inclusion criteria.

The criteria were again judged from top to bottom for each study; no further assessment was done on the subsequent criteria as soon as a criterion was not met. Any disagreements were resolved by discussion between the two reviewers; if no agreement could be reached, a third reviewer decided.

The third stage of inclusion related to the methodological assessment of the reviews. All reviews remaining after the second stage were assessed with the Overview Quality Assessment Questionnaire [83-85]. This instrument is one of the most frequently used appraisal instruments for systematic reviews in the biomedical literature [87], besides being one of the few found for which psychometric properties had been documented [88] and which had been found to meet several important criteria, such as construct validity, inter-observer reliability and coverage of the items in the QUORUM statement for reporting systematic reviews [89]. Scores on this instrument can vary from 1 (extensive flaws) to 7 (minimal flaws). Two reviewers performed this assessment independently. The mean of the scores of the two reviewers was computed and classified as the final quality judgment; in case the scores of the reviewers differed more than 2 points, reviewers discussed their assessments and came to a new joint score (this was only needed once, mean difference score was 0.91).

Only high quality reviews (= with mean scores of 5 (minor flaws) and above) were used for the data-extraction, as is proposed by Jadad et al. [90] and Peach [91], since it is known that low quality reviews may reach different conclusions than high quality reviews [92-94], and also to avoid false conclusions that are based on low quality evidence.

### Data-analysis and synthesis

Data were extracted about the applied in- and exclusion criteria for the primary studies, search strategies, studied interventions, time frame of the searches, selected outcomes, and selected patient populations, effects on patients, effects on health care use and costs.

As stated earlier two categorizations for the interventions were used to organize the data. Firstly, the rough two categories system of discharge interventions, divided in discharge preparation and discharge support interventions; secondly the categorization of Parker et al[81], who distinguish four broad classes of 'discharge arrangements': comprehensive discharge planning protocols, comprehensive geriatric assessment programmes, discharge support arrangements and educational interventions, all of which can be either generic or disease specific. The definitions of each category are already given in the Background of this article.

The outcomes were classified according to the research questions:

- The discharge status of patients: length of hospital stay, discharge destination, dependency at discharge

- The functioning of patients in the first 3 months after discharge: physical status, emotional status, social status, health status

- Health care services use and costs: readmissions, use of health care services post discharge, costs

Physical status concerns all measures about level of activities of daily living, self-care abilities, self efficacy or independence. Emotional status concerns all measures about the level of well-being of patients such as uncertainty, anxiety, depression, informational needs, mood or coping. Social status refers to the extent a patient is able to participate in normal social activities and relationships. Health status concerns symptom prevalence and burden, organ dysfunction, mortality, morbidity and physical complications. However, these categories are not always mutual exclusive, e.g. in the case where multi-dimensional quality of life measures were used.

Whether an outcome was regarded as a positive or a negative effect, was primarily based on the perspective and definitions used by the review authors. However, in general a shorter length of hospital stay, home as discharge destination, better physical, emotional and social functioning, better health status, less readmissions, less use of health care services and less costs were regarded as positive outcomes by the review authors, and consequently by us.

Data-analysis was done primarily by description of the interventions and by making cross-tables for the different interventions, populations and effects. No quantitative pooling was performed across the reviews.

Conclusions for the meta-review were based on the conclusions and results of meta-analyses presented in the reviews studied.

## Results

### Search and inclusion results

After duplicates had been removed, the searches in the different databases resulted in an initial set of 7442 references of potential interest. Initial sifting based on title and abstract reduced this set to 117 references. As said, the first reviewer carried out this process and a 10% random sample was also done independently by a second reviewer (crude agreement between reviewers was 99% with a kappa coefficient of 0.33). In addition, when a second reviewer checked a 10% random sample of the excluded references, discussion was only needed for two references and resulted in an exclusion-decision. The set of the 117 references, representing 108 reviews, was ordered full text for the second stage of the inclusion process. Two reviewers performed this second phase independently; agreement between reviewers in this phase was 79% with a kappa coefficient of 0.56. Discussion was needed for 23 references and agreement was subsequently reached. A set of 49 references, representing 41 reviews, finally proved to fulfil the inclusion criteria for type and content of study.

In the following stage, two reviewers independently assessed the remaining 41 reviews on their methodological quality, using the Overview Quality Assessment Questionnaire [83-85] proposed by Oxman. A mean of the two scores was computed and classified as the final quality judgment. Twenty-six reviews had a mean quality score lower than 5 and were excluded, while the remaining fifteen high quality reviews [79,81,95-107] advanced to the next stage of the review, for data-extraction and analysis.

**Figure 1:**
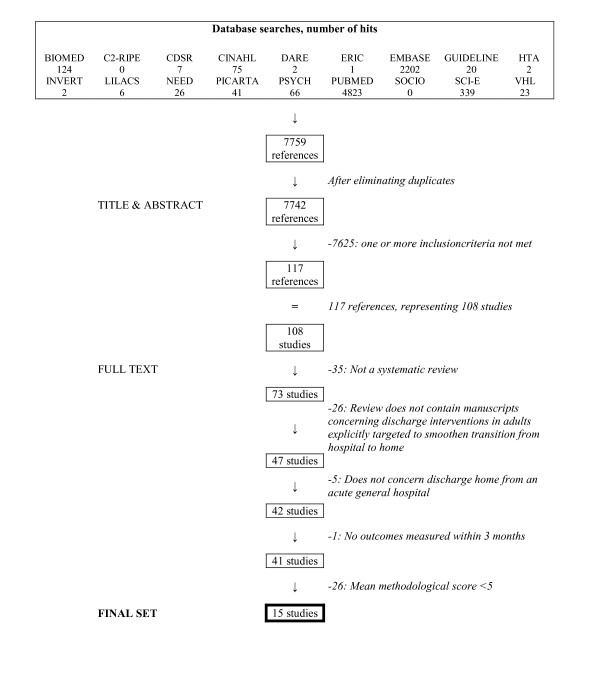
Flow diagram of the inclusion process.

The flow diagram of the inclusion process is shown in Figure 1. References of the studies excluded and the reason for exclusion can be found in Appendix 3 (see Additional file 3).

### Characteristics of the final 15 reviews

#### Publication date of the reviews and the journals in which they were published

All reviews included date from 2000 or later and five were published in 2004. The oldest reference included in a review dates from 1964 and the most recent one from 2004. Search periods for each review are shown in Table 1.

**Table 1:** Search periods in included reviews

**Review**	**Search period**
Cameron 2002	inception-2002
Cole 2001	1975–2000
Day 2004	1980–2003
Gwadry 2004	inception-2000
Handoll 2004	inception-2004
Hyde 2000	inception-1997
Kwan 2002	1975–2003
Outpatient Service Trialists (OST) 2003	inception-2001
Parker G 2000	1988–1999
Parker S 2002	inception-2001
Phillips 2004	inception-2003
Richards 2003	inception-2000
Shepperd 2001	inception-2001
Shepperd 2004	inception-2002
Teasell 2003	1995–2002

The reviews were published in eight different journals; six reviews [79,95,99,101,102,106] were published as a review in the Cochrane Database of Systematic Reviews.

#### Type and number of studies included in the reviews

Since all included reviews were focused on effectiveness, all reviews limited their inclusion criteria to comparative research designs. Seven reviews [81,98,102,104-107] were limited to randomized controlled trials only, while the other eight also included other comparative designs, such as quasi-randomized trials, non-randomized comparative studies and before-after designs. Two review authors [97,103] additionally searched for other reviews and guidelines and used these to reach their conclusions.

The fifteen reviews included a total of 265 different primary studies, the number of primary studies included in an individual review varying from 8 [98] to 71 [81]. Most (200 of the 265) of the primary studies were included only once in a review, with the exception of a few papers that were included in more than one review, extending to four inclusions for ten primary studies and with a maximum of five inclusions for two primary studies. A list of all primary studies included in one of the reviews can be found in Appendix 4 (see Additional file 4).

#### Aims of the reviews

The aims of the reviews included are all related to the effectiveness of discharge interventions, but there is a wide variation in what review authors describe as their objectives, as can be seen in Table 2.

**Table 2:** Aim of review, as worded by review-authors

**Review**	**Aim**
Cameron 2002	to examine the effectiveness and cost effectiveness of specialised multidisciplinary inpatient rehabilitation supervised by a geriatrician or rehabilitation physician compared with usual (orthopedic) care, for older patients with proximal femoral fracture
Cole 2001	to determine the impact of geriatric post-discharge services on mental state
Day 2004	to provide the evidence base on the effectiveness of specialist geriatric services for developing a sound practice framework
Gwadry 2004	to evaluate the effectiveness of multidisciplinary heart failure management programs on hospital admission rates
Handoll 2004	to evaluate the effects of different mobilisation strategies and programmes after hip fracture surgery
Hyde 2000	to investigate the effects of supported discharge after an acute admission in older people with undifferentiated clinical problems
Kwan 2002	to assess the effects of care pathways, compared with standard medical care, among patients with acute stroke who had been admitted to hospital. In particular we aimed to assess the effects on functional outcome, process of care, quality of life and the hospitalisation costs
OST 2003	to assess the effects of therapy-based rehabilitation services targeted towards stroke patients resident in the community within one year of stroke onset or discharge from hospital following stroke
Parker G 2000	to establish both the volume and strength of existing evaluative research on the costs, quality and effectiveness of different locations of acute, post- and subacute and rehabilitation care for older people
Parker S 2002	to test the following hypotheses: 1. There is an inadequate number of comparable rct's to allow a definitive analysis; 2. Hospital discharge process, outcome and cost-effectiveness can be improved through the use of a variety of interventions; 3. Some interventions are more effective than others; 4. there are priority areas for future research
Phillips 2004	to evaluate the effect of comprehensive discharge planning plus post-discharge support in patients with chronic heart failure on the rate of readmission, all cause mortality, length of stay, quality of life and medical costs
Richards 2003	to determine the effectiveness and costs of interventions intended to improve access to health and social care for older patients following discharge from acute hospitals
Shepperd 2001	to assess the effects of hospital at home compared with in-patient hospital care
Shepperd 2004	to determine the effectiveness of planning the discharge of patients moving from hospital
Teasell 2003	to assess the effectiveness of early supported discharge programs in the context of stroke rehabilitation

#### Patients of interest in the reviews

Some of the reviews included studies in which interventions targeted several or mixed patient populations, while others were restricted to studies with a specified patient group only (e.g. stroke patients, hip fracture patients, elderly or patients with heart failure). A combination was sometimes made of elderly patients and a specific medical condition. An overview is presented in Table 3.

**Table 3:** Patients of interest in the reviews

**Review**	**Several/mixed**	**Elderly**	**Stroke patients**	**Patients with hip or femur fractures**	**Patients with heart failure**
Cameron 2002				X	
Cole 2001		X			
Day 2004		X			
Gwadry 2004					X
Handoll 2004				X	
Hyde 2000		X			
Kwan 2002			X		
OST 2003			X		
Parker G 2000		X			
Parker S 2002		X			
Phillips 2004					X
Richards 2003		X			
Shepperd 2001	X				
Shepperd 2004	X				
Teasell 2003			X		
					

Total	2	6	3	2	2

#### Interventions studied in the reviews

As said, we used two categorization systems for the discharge interventions. For this paragraph only the results for the rough two categories system is presented. The grouping of the results by the second categorization system of Parker et al. [81] is presented in the more detailed section about the effectiveness of interventions later on.

According to the first system discharge interventions are classified into two groups, discharge preparation and discharge support interventions.

Some of the reviews included only studies that used interventions from the first group, others only included studies that used interventions from the second group, and a third category comprised reviews that included studies in which interventions from both groups had to be applied. The focus of the reviews is shown in Table 4.

**Table 4:** Focus of interventions in reviews

**Review**	**Focus on discharge preparation**	**Focus on discharge support/aftercare**
Cameron 2002	X	
Cole 2001		X
Day 2004	X	X
Gwadry 2004		X
Handoll 2004	X	X
Hyde 2000		X
Kwan 2002	X	
OST 2003		X
Parker G 2000		X
Parker S 2002	X	X
Phillips 2004	X	X
Richards 2003	X	X
Shepperd 2001		X
Shepperd 2004	X	
Teasell 2003		X
		

Total	8	13

Interventions included in discharge preparation reviews were care pathways, patient management schemes, specialized units (for stroke, hip fracture or geriatric patients for example), geriatric assessment and/or consultation, discharge coordinators, nurse specialists, educational interventions, intensified rehabilitation/(physio)therapy schemes, adjusting skill-mix of hospital professionals, and discharge plans.

Interventions included in the discharge support reviews were telephone follow-up, home visits, geriatric assessment and/or consultation, intensified post-discharge care (hospital at home), educational interventions and intensified rehabilitation/(physio)therapy schemes.

The interventions included in a particular review showed considerable heterogeneity in terms of what exactly was done, by whom it was done, the way it was done, the frequency with which it was done, and the duration of the intervention.

#### Control conditions in the reviews

Most reviews included studies in which patients in the control condition received usual care (according to the trial authors); other reviews included studies in which the different interventions were compared against each other (e.g. different rehabilitation/therapy schemes). The problem with the first category for all review authors was that the trial authors were not clear on what constituted 'usual care'.

#### Outcomes studied in the reviews

Some of the included reviews had well described primary outcomes that to had be described in the trials before they could be included, while others had no criteria at all with regard to outcomes as long as the studies dealt with the relevant intervention. Many of the outcomes, in both the primary studies and the reviews, lacked a clear definition, however, e.g. functional status or quality of life or mental state. In addition, different terms were used across primary studies and reviews for outcomes that are related or that are probably the same (e.g. physical status or functional status or ability in activities in daily living). Above this, even similar outcomes were measured with different (frequently not validated) instruments at different times post discharge, posing problems for the review authors in combining the effects across trials, but also in combining the results from reviews for this meta-review.

### Effectiveness of the discharge interventions

#### General picture

Although a statistically significant effect was occasionally found for a particular intervention on a particular outcome, most review authors reached no firm conclusions that the discharge interventions they studied were effective. Only two review authors [104,105] were firm in their conclusions. The conclusions as formulated by the authors are shown in Table 5, with formulations indicating no effects or inconclusive ones are shown in italics and formulations indicating firm conclusions are shown in bold typeface.

**Table 5:** Conclusions in included reviews

**Review**	**Conclusions**
Cameron 2002	The available trials had different aims, interventions and outcomes. Combined outcome measures (e.g. death or institutional care) *tended *to be better for patients receiving coordinated inpatient rehabilitation, but the results were heterogeneous and *not statistically significant*.
Cole 2001	There is *little evidence *that geriatric post-discharge services have an impact on the mental state of aged subjects.
Day 2004	This review generally supports the efficacy of specialist geriatric team services trained in geriatrics with a multidisciplinary collaborative focus undertaking assessment, rehabilitation and coordinated case management in community settings; both preventive care and supportive discharge in these settings *appear *to provide greater benefit over usual care; *however these benefits are not consistent *across all outcomes and although improvement in outcomes was often apparent, these were not always significant when compared with the comparison group. Efficacy of specialist geriatric services for inpatient settings was more diverse; this was due to the diversity of studies across the continuum of subacute, acute, postacute care in unit or ward settings with resulting heterogeneous outcomes and *only some of these outcomes showing significance *over usual care. With regard to day hospital and outpatient care, evidence for the efficacy of specialist geriatric services was lacking, with *no conclusive evidence *that the services are of greater benefit than usual care.
Gwadry 2004	This review *suggests *that specific heart failure targeted interventions significantly decrease hospital readmissions but do not affect mortality rates.
Handoll 2004	There is *insufficient evidence *from randomised trials to determine the effectiveness of the various mobilisation strategies that start either in the early post-operative period or during the later rehabilitation period
Hyde 2000	We believe that the results of this review provide reassurance that supporting discharge from hospital to home is of value. However, *important sources of uncertainty remain*, suggesting the need for further research. There was relative certainty that the proportion of those at home 6–12 months after admission is greater with supported discharge; this was associated with a consistent pattern of reduction in admission to long-stay care over the same period, without apparent increases in mortality. There was uncertainty about the effect of supported discharge on hospitalization. There were no rigorous data on functional status, patient and carer satisfaction and in consequence uncertainty about the overall effectiveness of supported discharge.
Kwan 2002	Use of stroke care pathways may be associated with positive and negative effects. Since most of the results have been derived from non-randomised studies, they are likely to be influenced by potential biases and confounding factors. There is currently *insufficient supporting evidence *to justify the routine implementation of care pathways for acute stroke management or stroke rehabilitation.
OST 2003	Therapy-based rehabilitation services targeted towards stroke patients living at home reduces the odds of a poor outcome and has a beneficial effect on a patient's ability to perform activities of daily living. *However, the evidence is derived from a review of heterogeneous interventions *and therefore further exploration of the interventions is justifiable.
Parker G 2000	Despite considerable recent development of different forms of care for older patients, *evidence about effectiveness and costs is weak*. However, evidence is also weak for longer-standing care models.
Parker S 2002	The evidence from these trials does not suggest that discharge arrangements have effects on mortality or length of hospital stay. This review supports the concept that arrangements for discharging older people from hospital can have beneficial effects on subsequent readmission rates. Interventions provided across the hospital-community interface, both in hospital and in the patient's home, showed the largest effects. *Evidence from RCT's is not available *to support the general adoption of discharge planning protocols, geriatric assessment processes or discharge support schemes as means of improving discharge outcomes.
Phillips 2004	**Comprehensive discharge planning plus postdischarge support for older people with chronic heart failure significantly reduced readmission rates and may improve health outcomes such as survival and quality of life without increasing costs.**
Richards 2003	The interventions provided and patient groups targeted by these services were heterogeneous. **There was, however, some evidence that services combining needs assessment, discharge planning and a method for facilitating the implementation of these plans were more effective than services that do not include the latter action**. The assessment of need may be insufficient in itself for the adequate provision of post-discharge care; needs assessment should be combined with a service that facilitates the implementation of care plans.
Shepperd 2001	*This review does not support the development of hospital at home services as a cheaper alternative to in-patient care*. Early discharge schemes for patients recovering from elective surgery and elderly patients with a medical condition may have a place in reducing the pressure on acute hospital beds, providing the views of the carers are taken into account. The evidence supporting hospital at home for patients recovering from stroke is *conflicting*. There is some evidence that admission avoidance schemes may provide a less costly alternative to hospital care.
Shepperd 2004	The impact of discharge planning on readmission rates, hospital length of stay, health outcomes and cost is *uncertain*.
Teasell 2003	Although *the majority of studies reported no statistically significant differences *in functional outcomes between the two groups, there was a reduction in hospital stays for patients receiving home-based therapy. These results suggest that patients with milder strokes who receive home-based therapies have similar functional outcomes to patients who receive traditional inpatient rehabilitation. There is strong evidence that high-level stroke patients discharged from an acute hospital unit can be rehabilitated in the community by an interdisciplinary stroke rehabilitation team without negative consequences. These patients attain similar functional outcomes compared to patients with equivalent stroke severity who receive inpatient rehabilitation. Community based programs also appear to reduce hospital length of stay, although we do not have evidence of an overall cost reduction. Although the effectiveness of early supported discharge programs for patients with moderate-to-severe deficits has not been well studied, limited evidence suggests that these patients are unsuitable candidates and should receive inpatient rehabilitation instead.

#### Effect of discharge interventions on discharge status

Length of stay was studied in nine reviews. The findings were inconclusive in four reviews [95,97,101,107], no significant differences were found in another four reviews [79,81,99,104] and one review [106] concludes that hospital length of stay was significantly shorter for 'hospital-at-home' interventions.

Discharge destination was studied in six reviews. Findings were inconclusive in one review [97] and no significant differences were found in four reviews [79,81,101,106], while one review [103] found a significant difference in the number of patients being discharged home when they were cared for at a stroke unit (based on three trials) but not when they were treated in hip units or geriatric units.

Dependency at discharge was studied in one review [101] and it was found, on the basis of two studies (one randomized and one non-randomized) that patients from the care pathway group were more dependent at discharge than the control group.

There is no evidence on the whole that discharge interventions have a positive impact at length of stay, discharge destination, or dependency at discharge.

#### Effect of discharge interventions on patient functioning after discharge

As was specified in the second research question, patient functioning after discharge was divided into four types: physical, emotional, social and health status. The effects of the discharge interventions are given for each of these, and subdivided according to the intervention classification scheme put forward by Parker et al. [81], in which there are four broad classes of discharge interventions: comprehensive discharge planning protocols, comprehensive geriatric assessment programmes, discharge support arrangements and educational interventions, all of which can be either generic or disease specific.

#### Effect of discharge interventions on physical status after discharge

The effect of interventions from the discharge planning category on physical status in the first 3 months after discharge was studied in three reviews [79,81,105]. Parker et al. [81] included RCT's only and found eight articles representing seven studies in which discharge planning was studied. All studies involved patients who had experienced discharge from an acute inpatient hospital stay and evaluated a comprehensive discharge protocol implemented by an individual who was either a specialist nurse, a social worker or an admitting clerk. The comprehensive discharge protocols were similar in design and were compared with usual discharge care. The protocols all had similar elements, including the assessment of patients, liaising with the patient's carer and other professionals to coordinate discharge and providing follow-up visits or telephone calls. Only two of the seven studies included in this part of the review considered outcomes related to physical function. No differences were found between experimental and control groups within 3 months after discharge. Richards and Coast [105] included five RCT's dealing with comprehensive discharge planning and came to the same conclusion as Parker et al. that no differences had been shown with regard to physical status. Shepperd et al. [79] included 11 RCT's, six of which presented data concerning physical status. Here too, no effects of discharge planning on physical status were found.

So, these three reviews discussing the impact of discharge planning on physical status after discharge are mutually consistent and all conclude that no effect of discharge planning has been demonstrated on physical status.

The effect of interventions from *the comprehensive geriatric assessment category *on physical status in the first 3 months after discharge was studied in three reviews on generic patient populations [81,97,105] and in one review on patients with femoral fractures [95]. Day and Rasmussen [97] conclude that measures of functional status were similar and showed no significant difference between the intervention and control groups. Parker et al. [81] point to the great variety of measures used to report physical function outcomes, making comparisons and pooling difficult. They say that the majority of studies appeared to have found no significant differences in the physical function outcomes of study patients and control patients over time. With regard to improvement in physical function over time, Parker et al. were able to calculate an odds-ratio over six studies and found a significant effect suggesting that the intervention was beneficial for physical functioning. These outcomes, however, were not measured within our stated timeframe of 3 months post discharge. Richards and Coast [105] included two studies in which functional status outcomes were measured within the 3 months after discharge and both found no differences. Finally, Cameron et al. [95] examined the effects of coordinated multidisciplinary inpatient rehabilitation by a geriatrician or rehabilitation physician compared with usual care for older patients with hip fracture, and they state that the available trials reviewed had a variety of aims, interventions and outcomes, making them difficult to combine. They conclude on the basis of nine trials that functional status did not improve consistently.

On the basis of these four reviews, therefore, it appears that comprehensive geriatric assessment has not been shown to have a positive impact on functional status within 3 months after discharge, in comparison with the control groups.

The effect of interventions from *the discharge support category *on physical status after discharge was studied in four generic [81,100,105,106] and two disease specific reviews [102,107], both in stroke patients. Hyde et al. [100] investigated the effects of supported discharge after an acute admission in older people with undifferentiated clinical problems, in which supported discharge was defined as actual additional support from any source provided to patients or their carers and commencing within one week of discharge following an acute admission. They included nine studies of which six provided data on functional status; however, there were no rigorous data on functional status that made pooled conclusions possible. Parker et al. [81] point to the wide range of types of intervention, varying from a single phone call after discharge to complex multidisciplinary interventions. They included twenty-eight controlled trials, nineteen of which reported on some aspect of physical functioning and eight of which were comparable enough to pool, but showed no significant effect on physical functioning. Richards and Coast [105] evaluated the effectiveness of organizational interventions that influence access to health and social care after discharge. They found considerable heterogeneity in the content of interventions and the selection of patient groups. They identified two trials that reported on functional status within 3 months of discharge, but both of these were inconclusive and did not suggest improvement. Shepperd et al. [106] assessed the effects of hospital-at-home compared with in-patient hospital care. Sixteen studies were included, eight of which measured functional status in elderly medical patients and two trials in patients following elective surgery. Although pooling was not possible, there were no indications that the functional status in the intervention groups was better at 3 months post discharge. The review of the Outpatient Service Trialists [102] considered interventions targeting stroke patients resident in the community setting. Fourteen trials were included, twelve of which involved patients who had experienced discharge from hospital; the trials included used a large number of heterogeneous outcome measures. It was found on the basis of twelve trials that patients who received therapy-based rehabilitation services after stroke were significantly more independent in personal activities of daily living than those patients who received no care or usual care. Most of the studies measured this outcome at 6 or 12 months after starting the therapy, however, and it is not clear how long this was after hospital discharge; no (pooled) data at 3 months post discharge are given in this review. Teasell et al. [107] studied the effectiveness of early supported discharge programs in stroke patients. Ten studies were included, eight of which reported some kind of functional outcome. None of these studies reported statistically significant differences between the treatment groups, indicating that functional outcome was not affected negatively or positively by the intervention. Pooling was not performed in this review.

On the basis of these six reviews, therefore, there are no indications that patients who receive supported discharge have a better physical status at 3 months after discharge than patients from the control groups.

The effect of *educational interventions *on physical status after discharge was covered by two reviews [81,105]. Parker et al. [81] studied if education interventions improved the outcome of discharge of elderly people from hospital; the interventions studied were described as mainly educational and could be limited to education or supplemented by other activities, such as home visits or telephone calls after discharge. Eleven studies were included, two of which contained data on physical status; one study found better results in the intervention group, but the other study found no effects. Richards et al. [105] studied discharge co-ordinator roles, which may incorporate educational interventions. Five studies were included, four of which contained data on physical status after discharge; none of these found significant differences between experimental and control groups.

On the basis of these two reviews, therefore, there are no clear indications that educational interventions have an effect on physical status after discharge.

Finally, Handol et al. [99] studied mobilisation strategies in hip fracture surgery patients. They conclude that there is insufficient evidence from randomized trials to determine the effectiveness of the various mobilization strategies.

In summary, we found no evidence base that discharge interventions have a positive impact on the physical status of patients after discharge. All the reviews included, however, had to contend with extensive heterogeneity in interventions, patient populations, and outcomes scales and times and with inadequate descriptions of control conditions, all of which made pooling difficult.

#### Effect of discharge interventions on emotional status after discharge

The effect of interventions from *the discharge planning category *on emotional status after discharge was studied in three reviews [79,81,105]. Parker et al. [81] found one discharge planning study that included emotional status outcomes, which stated that mean satisfaction scores changed little over time. Richards and Coast [105] included two studies that reported emotional function outcome within 3 months and both found no differences. Shepperd et al. [79] found two studies containing some kind of emotional function; one found some improvement on one parameter but not on two other emotional outcomes, while the second study failed to detect a difference.

On the basis of these three reviews, therefore, there are no indications that discharge planning affects emotional functioning after discharge.

The effect of interventions from *the comprehensive geriatric assessment category *on emotional status after discharge was covered by two reviews [81,105]. Parker et al. [81] found eight studies reporting on aspects of emotional status, only one of which reported a significantly greater improvement in cognitive scores in the intervention group than found in the controls. On the whole, however, the outcomes of intervention and control group patients were broadly similar, with no obvious benefit observable for patients undergoing comprehensive geriatric assessment. Richards and Coast [105] included three studies in which some emotional outcome was reported within 3 months after discharge, but none of the three found differences between intervention and control groups.

On the basis of these two reviews, therefore, there are no indications that comprehensive geriatric assessment has a positive impact on emotional status after discharge.

The effect of interventions from *the discharge support category *on emotional status after discharge was studied in four generic reviews [81,96,105,106] and in one disease specific review [102]. Cole [96] found eleven trials reporting emotional status outcomes after geriatric post-discharge services, with the type of intervention and the type of emotional status outcomes varying from one study to the next. Emotional status outcomes included depression, morale, life satisfaction, contentment, emotional function, self perceived health or cognition. Three trials reported small effects and eight reported no effect. Parker et al. [81] found nine trials reporting on emotional functioning, including cognitive function (five trials) and measures of anxiety (three trials) or depression (two trials). They state that emotional status is measured in a variety of ways and in multiple domains, making interpretation or synthesis across studies problematic, and that in general, these measures remained unchanged between intervention and control groups. In addition, Parker et al. refer to sixteen trials measuring dimensions of quality of life, which may incorporate emotional status. Here too, they found many different instruments and that the data on the whole did not suggest that discharge support arrangements had a major impact on the quality of life of subjects when compared to controls. Finally, Parker et al. refer to six trials in which satisfaction was recorded. Four of the trials suggested some increased satisfaction with the service provided, but the data were neither consistently nor reliably reported. Richards and Coast [105] included two trials in this category; neither of which found differences in emotional status outcomes. To the extent that early discharge can be regarded as 'discharge support', Shepperd et al. [106] found eight trials involving medical patients in which some dimensions of psycho-social well-being or quality of life were measured. Six failed to detect a difference between intervention groups and control groups, while two studies reported more psycho-social dysfunction for the intervention group. Two trials involving surgery patients were included and failed to detect differences in this dimension. With regard to patient satisfaction, there was a mixed and ambivalent picture, but satisfaction tended to be higher in the hospital-at-home groups. No pooling was possible on these variables. The Outpatient Service Trialists [102] pooled results from five studies of quality of life in stroke patients and found no significant difference between experimental groups and control groups, which also applied to the findings of six studies in which mood/distress was measured.

On the basis of these five reviews, therefore, there are no indications that discharge support interventions enhance emotional functioning after discharge.

The effect of *educational interventions *on emotional status after discharge was covered by two reviews [81,105]. Parker et al. [81] found three studies of educational interventions that investigated the effect on emotional function; pooling was impossible and the effects were mixed: one study found no differences except for increased self-efficacy for walking; the second study had no measurements after discharge, and the third study, in which an education intervention in hospital was supported with extensive telephone follow-up after discharge, showed significantly lower levels of anxiety and a higher level of knowledge at 6 weeks after discharge. They also found four studies that considered the effect of educational interventions on adherence to medication advice, in which different measures were used to assess adherence, including tablet counts, self-reports of compliance and knowledge of medication regimens. All but one of these studies showed some improvements in adherence to medication or knowledge and it is concluded that more intensive interventions appear to be relatively effective, but that brief counselling or education is of little effect. Richards and Coast [105] studied discharge coordinator roles, which may incorporate educational interventions. Five studies were included, three of which contained data on emotional status after discharge, and none of these found significant differences between experimental groups and control groups.

On the basis of these two reviews, therefore, it appears that educational interventions might have some effect on aspects of emotional status after discharge, on knowledge and medication adherence, but the results of the reviews are not straightforward and the effects seem to depend on the dose and format of the educational interventions.

In summary, discharge interventions appear to have no effect, or only a very limited one, on the emotional status after discharge.

#### Effect of discharge interventions on social status after discharge

Data on the effect of interventions from the *comprehensive discharge planning category *on social status in first 3 months after discharge were found in one review [105]. Richard and Coast [105] included five studies with comprehensive discharge planning coordinators, and none found differences in social support experienced.

The effect of interventions from the *postdischarge support category *on social status was found in three reviews [81,103,106]. Parker G et al. [103] report about three trials in which there was no difference in patients at home at 3 months. Parker S et al. [81] found no statistical difference between experimental groups and control groups in number of patients being at home, based on six trials that measured this within first six months. On the basis of three trials, Shepperd et al. [106] found a significantly larger number of patients from the hospital-at-home group being at home at 6 weeks.

No reviews discussed effects of interventions from *geriatric assessment category *or of educational interventions on social status.

Finally, Handoll et al. [99] mention one small trial, in which no difference was found in loss of social independence between intensive physical training and placebo activities started post discharge.

In summary, there is a little bit of evidence, based on one review [106], that patients treated in hospital-at-home interventions more frequently remain at home than the control patients. The other four reviews, however, found no differences with regard to social status after discharge.

#### Effect of discharge interventions on health status after discharge

*Mortality *is certainly the outcome that has been looked at most frequently in the reviews, regardless the focus of the interventions. Most of the reviews (and the underlying trials), however, looked at mortality over more extended periods of time than the 3 months that are of interest in this meta review; mortality was mostly measured at 6 or 12 months. Twelve reviews [79,81,95,98-106] found no significant differences in mortality and only Day and Rasmussen [97] conclude that stroke units showed significant benefits in terms of mortality reduction, but do not specify the trials on which this conclusion is based.

The four reviews [95,97,99,106] in which *morbidity or complications *after discharge was studied and that were able to include trials, found no significant differences.

In summary, we found no firm evidence that discharge interventions have a positive impact on health status of patients after discharge.

#### Effect of discharge interventions on health care use after discharge and costs

*Readmissions *were measured in eleven reviews, but the measurement period was frequently 6 or 12 months and not the 3 months that is of interest for this meta-review.

Seven reviewers [79,95,97,100,102,103,105] are inconclusive about the effect of discharge interventions on readmission rates. One reviewer [106] found no statistically significant difference for patients in a hospital-at-home intervention. Three reviews [81,98,101] found a positive effect on readmissions. Parker et al. [81] reviewed four types of discharge interventions and conclude that when all interventions groups are taken together, the patients in the intervention groups have a significant lower risk of being readmitted and this was more marked among interventions provided both at hospital and at home. In the subgroups they did not find a significant difference for discharge planning activities, discharge support or geriatric assessment but they did find a significant difference in favour of patients receiving some kind of educational intervention. This is congruent with the positive finding of Gwadry et al. [98], that patients receiving a heart failure management program are less frequently readmitted. Finally, Kwan and Sandercock [101] found fewer readmissions for patients that were cared for in a stroke care pathway.

Three reviews [81,105,106] had included and discussed trials relating to the *use of services after discharge *and all were inconclusive on this subject.

All reviewers comment on the variety of ways that *costs*, cost-benefit and cost-effectiveness were measured in the trials, making synthesis difficult. Costs are also largely dependent on the organization of health care in an individual country, making cross-country synthesis difficult.

With this in mind, all reviewers [79,81,95,97,99,101,103,105-107] who report on costs are inconclusive about the impact of discharge interventions on costs.

In summary, there is little evidence that discharge interventions have an impact on health care use after discharge, or on costs, except that educational interventions may reduce readmissions in heart failure patients.

#### Effects of discharge interventions in specific patient groups

Three reviews [101,102,107] focused on *stroke patients *and compared several care delivery models and rehabilitation services. The main aim of this group of studies was more on the post-discharge period than on the discharge itself. Kwan and Sandercock [101] conclude that stroke care pathways may be associated with positive and negative effects and that there is currently insufficient evidence to justify the implementation of care pathways for acute stroke management or stroke rehabilitation. The Outpatient Service Trialists [102] conclude that therapy-based rehabilitation services targeting stroke patients living at home reduce the odds of a poor outcome and have a beneficial effect on a patient's ability to perform activities of daily living. They warn, however, that the evidence is derived from heterogeneous interventions and further exploration of the interventions is justifiable as a result. Teasell et al. [107] conclude that there is strong evidence that high-level stroke patients discharged from an acute hospital unit can be rehabilitated in the community by an interdisciplinary stroke rehabilitation team without negative consequences, and that community based programs also appear to reduce hospital length of stay.

Two reviews [95,99] concentrated on *patients with fractures*. Cameron et al. [95] state that the available RCT's had different aims, interventions and outcomes and were of poor to moderate quality, thus allowing only tentative conclusions. Combined outcome measures (e.g. death or institutional care) tended to be better for patients receiving coordinated inpatient rehabilitation, but the results were heterogeneous and not statistically significant. Handoll et al. [99] conclude that there is insufficient evidence to determine the effectiveness of the various mobilization strategies that start either in the early post-operative period or during the later rehabilitation period'.

Two reviews [98,104] concentrated on *cardiac patients*. Gwadry et al. [98] evaluated the effectiveness of multidisciplinary heart failure management programs on hospital readmission rates and found a significant decrease in these rates. Phillips et al. [104] also conclude that comprehensive discharge planning plus postdischarge support for older people with chronic heart failure significantly reduced readmission rates, and may improve health outcomes such as survival and quality of life without increasing costs. Based on above two reviews, it appears that readmissions in heart failure patients can be reduced by some kind of intervention.

## Discussion

We found more than forty systematic reviews of discharge interventions, fifteen of which scored highly on methodological quality. Our conclusions on the basis of these fifteen reviews, is that there is only limited evidence for the positive impact of discharge interventions. We found a few indications that discharge interventions may be effective. Three reviews [81,104,105] state that effects are mainly observed when interventions from the discharge planning and discharge support side were combined across the hospital-home interface. In addition, two reviews [81,105], appear to show that educational interventions might have some effect on aspects of the emotional status after discharge, on knowledge and medication adherence.

The limited evidence about effectiveness of discharge interventions may be due to the heterogeneity of several aspects which review authors had to deal with. All review authors were confronted with heterogeneity in interventions, control conditions, patient populations, outcome definition, methods of outcome measurement, outcomes assessment times, and in other aspects. This heterogeneity made it difficult for the review authors to synthesize the results of the underlying trials and this mostly led to inconclusive conclusions.

It may be that discharge interventions do have an impact, but that measurements of outcomes are not reliable or not sensitive enough. There is also a possibility that discharge interventions do have an effect, but that this is not longstanding and can no longer be measured at the time of the outcome assessments. On the other hand, there is a possibility that effects of discharge interventions only show up after the three months after discharge to which we had limited the meta-review. There is no good theoretical base for either option, however, whether very short-term or very long-term. It may also be that patients in control conditions received more care than is suggested by the term 'usual care', which was mostly ill-defined. Another possibility is that discharge interventions are only working in specific subgroups of patients, or that discharge interventions are only effective in higher intensities.

On the other hand, we did find a few indications that discharge interventions may be effective. Three reviews [81,104,105] state that effects are seen in particular when interventions from the discharge planning and discharge support side were combined across the hospital-home interface. If discharge planning interventions are to be effective, they should have to be combined with discharge support interventions and vice versa. In addition, two reviews [81,105] appear to show that educational interventions might have some effect on parts of the emotional status after discharge, on knowledge and medication adherence, but the results of the reviews are not straightforward and effects appear to be dependent on the quantity and format of the educational interventions.

We also had one review [101], however, in which it was concluded that the effect of a discharge intervention was in the opposite direction to what had been expected, since they found that patients from the care pathway group were more dependent at discharge then the control group.

An interesting finding in this meta-review is that only a few trials were included in more than one review, although all included reviews had a related topic of research and all applied sensitive methods to find the primary research. It is possible that the final inclusion sets of each review differ due to different focuses of each review, what causes differences in search strategies and inclusion criteria. However, the question remains that, if a meta-review were to be done on the data from all of the 265 primary studies included in one of the reviews, whether this would lead to conclusions similar to those we have now obtained.

It could be argued that this meta-review does not give a complete picture of the state of art, because there are many more reviews on discharge interventions than were included in this review. Inclusion of reviews of a lower methodological quality would certainly have added some information, but these findings are less reliable in our opinion and would have led to more uncertainty. Moreover, we believe it gives cause for concern that we excluded more than half of the reviews found, solely on the basis of the suboptimal methodological quality of the systematic review.

It could also be argued that this meta-review is not up to date, since it was limited to reviews dated pre-2005. There may be more recent systematic reviews with conclusions different to those presented here. When a quick search for recent reviews was made in PUBMED and CINAHL in November 2006, however, and without a formal inclusion process applied, we found no indications that this would have altered our conclusions. There is a review, for example, that reaches firm conclusions that implementing a telemanagement program directed by an advanced practice nurse after hospital discharge decreases the costs and frequent rehospitalizations associated with heart failure and improves the patient's quality of life [108], but also a review that states that the evidence, as it stands at present, raises a number of issues about current hospital discharge policy [109], one that concludes that hospital-based case management did not reduce length of hospital stay or readmissions in adult inpatients [110], and another review that states that there was inconclusive evidence about the effects of telephone follow-up after discharge [111].

From a research point of view, many challenges remain in proving the (in)effectiveness of discharge interventions: better designs, better instruments, better descriptions of interventions and control conditions, and many more.

Challenges also remain for reviewers in applying strategies to find all available research data, but also in finding methods of synthesizing results containing a high degree of heterogeneity. Questions remain when reviews are comparable enough to allow synthesizing the results in the way it was done in this meta-review; maybe the umbrella concept of 'discharge interventions' is too broad to endeavour synthesizing by means of a review of systematic reviews already dealing with vast heterogeneity.

Finally, challenges remain for meta-reviewers in developing methods for synthesizing results of the relevant reviews available. The methodology for doing systematic reviews is well developed nowadays and well described for instance in the Cochrane handbook for reviewers, but a well founded methodology and rationale for performing a systematic review of reviews is currently lacking, especially with regard to the ways of synthesizing data. Such methodology is hardly needed due to rapidly growing amount of published reviews on a same or related topic. In this respect, we advise to follow closely the ongoing work of the recently started Cochrane Umbrella Reviews Working Group.

From a practical point of view, this meta-review is rather disappointing, since there is only limited evidence to give directions to how health care professionals and organisations can adopt discharge planning or discharge support interventions. Usual care seems to be equally as effective or ineffective as discharge interventions. Post-discharge problems continue to be an important issue, however, which means that professionals and organisations must consider ways of preventing, easing or solving post-discharge problems.

## Conclusion

Based on fifteen high quality systematic reviews, there is some evidence that some interventions, particularly those with educational components and those which combine pre-discharge and post-discharge interventions, may have a positive impact but there is, on the whole, limited summarized evidence that discharge planning and discharge support interventions have a positive impact on patient status at hospital discharge, on patient functioning after discharge, or on health care use after discharge and costs.

## Competing interests

The author(s) declare that they have no competing interests.

## Authors' contributions

All authors made a substantive contribution to all parts of this study. PM initiated and designed the study, developed and performed literature searches, was the primary responsible person for study inclusion and quality assessment, did the data-extraction and synthesis and made the first draft of this manuscript. AF en EP were involved in commenting the study design, participated in study inclusion and quality assessment, and contributed substantively in drafting and revising the manuscript.

All authors read and approved the final manuscript.

## Pre-publication history

The pre-publication history for this paper can be accessed here:



## Supplementary Material

Additional file 1 Appendix 1: Data sources. The table shows all the data sources that were searched Click here for file

Additional file 2 Appendix 2: Search strategies. The table shows the applied search strategies for each data source Click here for file

Additional file 3 Appendix 3: Excluded studies and reason for exclusion. The data show the references that were excluded and the reason why Click here for file

Additional file 4 Appendix 4: List of references of primary studies included in one of the reviews. The data show the references of the primary studies included in one of the reviews Click here for file
