# Supplementary material for: Interventions aimed at reducing problems in adult patients discharged from hospital to home: a systematic meta-review
Source: BMC Health Serv Res. 2007 Apr 4;7:47. doi: 10.1186/1472-6963-7-47 (PMC1853085; doi:10.1186/1472-6963-7-47)
Supplement: Additional file 1 — Appendix 1: Data sources. The table shows all the data sources that were searched [file 1472-6963-7-47-S1.doc]

**Appendix 1: Data sources**

For finding reviews, following literature databases were searched:

1. BiomedCentral (BIOMED)
2. Campbell Collaboration Reviews of Interventions, and Policy Evaluations (C2-RIPE)
3. Cochrane Database of Systematic Reviews (CDSR)
4. Cumulative Index of Nursing and Allied Health professions Literature (CINAHL)
5. Database of Abstracts of Reviews of Effects (DARE)
6. Education Resources Information Center (ERIC)
7. Excerpta Medica online (EMBASE)
8. Health Technology Assessment Database (HTA)
9. Index van de Nederlandstalige VERpleegkundige Tijdschriftliteratuur (Dutch nursing literature index) (INVERT)
10. Latin American and Caribbean Health Sciences Literature (LILACS)
11. NHS Economic Evaluation Database (NEED)
12. Picarta (Dutch library system) (PICARTA)
13. Psychological Abstracts Database (PSYCHINFO)
14. PUBMED
15. Sociological Abstracts Database (SOCIOFILE)
16. the Combined Social Science Citation Index/ Science Citation Index Expanded/ Arts & Humanities Citation Index (SCI-E)
17. Virginia Henderson Library (VHL)

For finding systematic reviews as part of a guideline following websites were searched:

1. Agency for Healthcare Research and Quality ([www.ahcpr.gov](http://www.ahcpr.gov/))
2. British Columbia Council on Clinical Practice Guidelines (<http://www.hlth.gov.bc.ca/msp/protoguides/>)
3. Centre for Health Services Research (<http://www.ncl.ac.uk/pahs/research/services/publications/guide/index.htm>)
4. Centre for Reviews and Dissemination (<http://144.32.150.197/scripts/WEBC.EXE/NHSCRD/start>)
5. Dutch Institute for Healthcare Improvement ([www.cbo.nl](http://www.cbo.nl/))
6. eGuidelines (<http://www.eguidelines.co.uk/>)
7. Guidelines Advisory Committee (http://www.gacguidelines.ca/)
8. Guidelines International Network ([www.g-i-n.net](http://www.g-I-n.net/) )
9. Haute Autorité de Santé (<http://www.anaes.fr/>)
10. Joanna Briggs Institute ([www.joannabriggs.edu.au](http://www.joannabriggs.edu.au/))
11. Deutsches **Netzwerk für Qualitätsentwicklung in der Pflege** ([www.dnqp.de](http://www.dnqp.de/) )
12. Leitlinien (http://www.leitlinien.de/)
13. National electronic Library for Health (<http://www.nelh.nhs.uk/>)
14. National Guideline Clearinghouse ([www.guidelines.gov](http://www.guidelines.gov/))
15. National Health an d Medical Research Council (<http://www7.health.gov.au/nhmrc/publications/cphome.htm> )
16. National Institute for Clinical Excellence (<http://www.nice.org.uk/>)
17. New Zealand Guidelines Group ([www.nzgg.org](http://www.nzgg.org/))
18. New Zealand Health Technology Assessment Clearing House for Health Outcomes and Health Technology Assessment (<http://nzhta.chmeds.ac.nz/>)
19. NHS Health Technology Assessment Programme (<http://www.hta.nhsweb.nhs.uk/>)
20. Registered Nurses Association of Ontario ([www.rnao.org](http://www.rnao.org/))
21. Royal College of Nursing ([www.rcn.org.uk](http://www.rcn.org.uk/) )
22. Scottish Intercollegiate Guidelines Network ([www.sign.ac.uk](http://www.sign.ac.uk/))
23. Sumsearch (<http://sumsearch.uthscsa.edu/>)
24. Trip database ([http://www.tripdatabase.com](http://www.tripdatabase.com/))
25. World Health Organization (<http://www.who.int/site/en/>)
