# Supplementary material for: Interventions aimed at reducing problems in adult patients discharged from hospital to home: a systematic meta-review
Source: BMC Health Serv Res. 2007 Apr 4;7:47. doi: 10.1186/1472-6963-7-47 (PMC1853085; doi:10.1186/1472-6963-7-47)
Supplement: Additional file 2 — Appendix 2: Search strategies. The table shows the applied search strategies for each data source [file 1472-6963-7-47-S2.doc]

# Appendix 2: Search strategies

| **Database** | **Interface** | **Search strategy** |
| --- | --- | --- |
| PUBMED | [www.pubmed.gov](http://www.pubmed.gov/) | ("patient discharge"[MeSH Terms] OR ((patient* OR client* OR consumer* OR recipient* OR subject*) AND discharg*) OR "hospital discharge"[tw] OR "aftercare"[MeSH Terms] OR aftercare [tw] OR "continuity of patient care"[MeSH Terms] OR “continuity of patient care” OR “continuity of care” OR post?hospital* OR post?discharge) AND (review [pt] OR ((meta-analysis [pt] OR meta-anal* [tw] OR metaanal* [tw] OR (quantitativ* review* [tw] OR quantitative* overview* [tw] ) OR (systematic* review* [tw] OR systematic* overview* [tw]) OR (methodologic* review* [tw] OR methodologic* overview* [tw]) OR (review [pt] AND medline [tw]) OR systematic [sb]) OR (guideline [pt] OR practice guideline [pt] OR health planning guidelines [mh] OR consensus development conference [pt] OR consensus development conference, nih [pt] OR consensus development conferences [mh] OR consensus development conferences, nih [mh] OR guidelines [mh] OR practice guidelines [mh] OR (consensus [ti] AND statement [ti])))) AND "humans"[MeSH Terms] AND ("1994"[PDAT] : "2004"[PDAT]) |
| BIOMED | [www.biomedcentral.com](http://www.biomedcentral.com/) | [(discharge [TIAB] OR aftercare [TIAB] OR post?discharge [TIAB] OR "continuity of care" [TIAB])](http://www.biomedcentral.com/search/results.asp?txtSearch1=(discharge+%5BTIAB%5D+OR+aftercare+%5BTIAB%5D+OR+post%3Fdischarge+%5BTIAB%5D+OR+"continuity+of+care"+%5BTIAB%5D)&drpFromDate=1997&drpToDate=2004&drpAddedInLast=0&drpArticleType= ) |
| C2-RIPE | [www.campbellcollaboration.org](http://www.campbellcollaboration.org/) | discharge OR aftercare |
| CDSR | Wiley | ("patient discharge" OR "discharge planning" OR aftercare OR "hospital discharge" OR "continuity of care" OR "continuity of patient care" OR post?hospital OR post?discharge) in Record Title, from 1994 to 2004 in The Cochrane Database of Systematic Reviews |
| CINAHL | EBSCO | ((((((MH "Patient Discharge")) or (MH "Patient Discharge+")) or (MH "Patient Discharge Education")) or (MH "Discharge Planning+")) Or discharge planning Or aftercare Or “continuity of care”) AND (( ((MH "Systematic Review") or (MH "Cochrane Library") or (MH "Literature Review+")) ) or literature review or meta-analysis) |
| DARE | Wiley | ("patient discharge" OR "discharge planning" OR aftercare OR "hospital discharge" OR "continuity of care" OR "continuity of patient care" OR post?hospital OR post?discharge) in Record Title, from 1994 to 2004 in Database of Abstracts of Reviews of Effects |
| ERIC | EBSCO | ("patient discharge" OR "discharge planning" OR aftercare OR "continuity of care" OR "continuity of patient care" OR "hospital discharge") AND (review OR meta-analysis)  *limited to journal articles* |
| EMBASE | www.embase.com | (('patient'/exp OR 'patient') AND discharge OR ('hospital'/exp OR 'hospital') AND discharge OR continuity AND of AND care OR continuity AND of AND ('patient'/exp OR 'patient') AND care OR discharge AND ('planning'/exp OR 'planning') OR ('aftercare'/exp OR 'aftercare') OR post?discharge OR post?hospital AND [article]/lim AND [embase]/lim AND [1994-2004]/py) AND (('literature'/exp OR 'literature') AND ('review'/exp OR 'review') OR ('literature'/exp OR 'literature') AND overview OR systematic AND review* OR systematic AND overview* OR ('meta analysis'/exp OR 'meta analysis') OR meta?analy* OR guideline* OR [review]/lim AND [article]/lim AND [embase]/lim AND [1994-2004]/py) |
| HTA | Wiley | ("patient discharge" OR "discharge planning" OR aftercare OR "hospital discharge" OR "continuity of care" OR "continuity of patient care" OR post?hospital OR post?discharge) in Record Title, from 1994 to 2004 in Health Technology Assessment Database |
| INVERT | [www.invert.be](http://www.invert.be/) | (ontslag OR ontslagmanagement OR ontslagvoorbereiding OR ziekenhuisontslag OR ontslagprocedures OR postontslag OR postontslagproblemen OR continuiteit OR nazorg) AND literatuurstudie  *limited 1994-2004* |
| LILACS | [http://bases.bireme.br](http://bases.bireme.br/) | discharge [Palavras] or aftercare [Palavras] and review [Tipo de publicação]  *limited 1994-2004* |
| NEED | Wiley | ("patient discharge" OR "discharge planning" OR aftercare OR "hospital discharge" OR "continuity of care" OR "continuity of patient care" OR post?hospital OR post?discharge) in Record Title, from 1994 to 2004 in NHS Economic Evaluation Database |
| PICARTA | [www.picarta.nl](http://www.picarta.nl/) | ((ontslagvoorbereiding of ontslaggesprek of "continuiteit van zorg") en (ziekenhuis of hospitaal)) en JVU 1994-2004  *limited to articles* |
| PSYCHINFO | Webspirs | ((explode "Discharge-Planning" in MJ,MN) or (explode "Facility-Discharge" in MJ,MN) or (explode "Hospital-Discharge" in MJ,MN) or (explode "Aftercare-" in MJ,MN) or (explode "Client-Transfer" in MJ,MN) or (discharge planning) or (post?discharge) or (post?hospital) or (continuity of patient care) or (continuity of care) or (aftercare)) and (((DT:PSYI = REVIEW) or (explode "Literature-Review" in MJ,MN) or (systematic review)or (meta?analysis)or (literature study) or (literature review)) or ((explode "Treatment-Guidelines" in MJ,MN) or (guideline*) or (consensus development))) and (PY:PSYI = 1994-2004) |
| PUBMED | [www.pubmed.gov](http://www.pubmed.gov/) | ("patient discharge"[MeSH Terms] OR ((patient* OR client* OR consumer* OR recipient* OR subject*) AND discharg*) OR "hospital discharge"[tw] OR "aftercare"[MeSH Terms] OR aftercare [tw] OR "continuity of patient care"[MeSH Terms] OR “continuity of patient care” OR “continuity of care” OR post?hospital* OR post?discharge) AND (review [pt] OR ((meta-analysis [pt] OR meta-anal* [tw] OR metaanal* [tw] OR (quantitativ* review* [tw] OR quantitative* overview* [tw] ) OR (systematic* review* [tw] OR systematic* overview* [tw]) OR (methodologic* review* [tw] OR methodologic* overview* [tw]) OR (review [pt] AND medline [tw]) OR systematic [sb]) OR (guideline [pt] OR practice guideline [pt] OR health planning guidelines [mh] OR consensus development conference [pt] OR consensus development conference, nih [pt] OR consensus development conferences [mh] OR consensus development conferences, nih [mh] OR guidelines [mh] OR practice guidelines [mh] OR (consensus [ti] AND statement [ti])))) AND "humans"[MeSH Terms] AND ("1994"[PDAT] : "2004"[PDAT]) |
| SOCIOFILE | CSA Internet Database Service | KW=(review or literature or meta-analysis) and (KW=aftercare or KW=(continuity of patient care) or KW=(continuity of care) or KW=(discharge planning)) and KW=hospital *limited 1994-2004* |
| SCI-E | ISI Web of Knowledge | TS=(((discharge planning) OR (patient discharge) OR (hospital discharge) OR (post?discharge) OR (aftercare) OR (continuity of care) OR (continuity of patient care)) AND ((systematic review) OR (systematic overview) OR (literature review) OR (literature overview) OR (literature study) OR (metaanalysis) OR (meta-analysis) OR (meta?anal*) OR (guideline)))  *DocType=All document types; Language=All languages; Databases=SCI-EXPANDED, SSCI, A&HCI; Timespan=1994-2004* |
| VHL | [www.stti.org/VirginiaHendersonLibrary/](http://www.stti.org/VirginiaHendersonLibrary/) | Discharge planning (in Study Title) OR aftercare (in Study Title)  *Timespan=1994-2004* |
