# Supplementary material for: Interventions aimed at reducing problems in adult patients discharged from hospital to home: a systematic meta-review
Source: BMC Health Serv Res. 2007 Apr 4;7:47. doi: 10.1186/1472-6963-7-47 (PMC1853085; doi:10.1186/1472-6963-7-47)
Supplement: Additional file 3 — Appendix 3: Excluded studies and reason for exclusion. The data show the references that were excluded and the reason why [file 1472-6963-7-47-S3.doc]

# Appendix 3: Excluded studies and reason for exclusion

| Reference | **Reason for exclusion** |
| --- | --- |
| Akner G, Boreus L, Elmstahl S, Eriksson S, Granerus A, Lernfelt B *et al*.: *Geriatric Care and Treatment. A systematic compilation of existing scientific literature*. Stockholm: Swe­dish Council Technology Assessment in Health Care; 2003. | Not a systematic review |
| Ali W, Rasmussen P: **What is the evidence for the effectiveness of managing the hospital/community interface for older people? A critical appraisal of the literature.** *NZHTA REPORT* 2004, **7:** 1-146. | Mean methodological score <5 |
| Andersen HE, Jurgensen KS, Boysen G: **[Intervention for apoplexy patients discharged from hospital. Psychosocial support: a literature review].** *Ugeskr Laeger* 2001, **163:** 1250-1254. | Mean methodological score <5 |
| Andersen HE, Jurgensen KS, Boysen G: **[Intervention for apoplexy patients discharged from hospital. Physical training: a literature review].** *Ugeskr Laeger* 2001, **163:** 1255-1259. | Mean methodological score <5 |
| Anderson C, Ni MC, Brown PM, Carter K: **Stroke rehabilitation services to accelerate hospital discharge and provide home-based care: an overview and cost analysis.** *Pharmacoeconomics* 2002, **20:** 537-552. | Mean methodological score <5 |
| Avenell A, Handoll HH: **Nutritional supplementation for hip fracture aftercare in the elderly.** *Cochrane Database Syst Rev* 2004, CD001880. | Review does not contain manuscripts concerning discharge interventions explicitly targeted to smoothen transition from hospital to home |
| Balinsky W, Muennig P: **The costs and outcomes of multifaceted interventions designed to improve the care of congestive heart failure in the inpatient setting: a review of the literature.** *Med Care Res Rev* 2003, **60:** 275-293. | Mean methodological score <5 |
| Basset JA, Tapuska B, Ford P, Barbe J: **Enteral nutrition: hospital to home a collaborative approach to education and care.** *Perspectives (Montclair)* 1997, **21:** 2-6. | Not a systematic review |
| Benbassat J, Taragin M: **Hospital readmissions as a measure of quality of health care: advantages and limitations.** *Arch Intern Med* 2000, **160:** 1074-1081. | Mean methodological score <5 |
| Beswick AD, Rees K, Griebsch I, Taylor FC, Burke M, West RR *et al*.: **Provision, uptake and cost of cardiac rehabilitation programmes: improving services to under-represented groups.** *Health Technol Assess* 2004, **8:** iii-x, 1. | Review does not contain manuscripts concerning discharge interventions explicitly targeted to smoothen transition from hospital to home |
| Bourbonniere M, Kagan SH: **Nursing intervention and older adults who have cancer: specific science and evidence based practice.** *Nurs Clin North Am* 2004, **39:** 529-543. | Mean methodological score <5 |
| Bours GJ, Ketelaars CA, Frederiks CM, Abu-Saad HH, Wouters EF: **The effects of aftercare on chronic patients and frail elderly patients when discharged from hospital: a systematic review.** *J Adv Nurs* 1998, **27:** 1076-1086. | Mean methodological score <5 |
| Bright D, Walker W, Bion J: **Clinical review: Outreach - a strategy for improving the care of the acutely ill hospitalized patient.** *Crit Care* 2004, **8:** 33-40. | Review does not contain manuscripts concerning discharge interventions explicitly targeted to smoothen transition from hospital to home |
| Britton A, Russell R: **Multidisciplinary team interventions for delirium in patients with chronic cognitive impairment.** *Cochrane Database Syst Rev* 2000, CD000395. | Review does not contain manuscripts concerning discharge interventions explicitly targeted to smoothen transition from hospital to home |
| Bull MJ: **Discharge planning for older people: a review of current research.** *Br J Community Nurs* 2000, **5:** 70-74. | Mean methodological score <5 |
| Campbell H, Hotchkiss R, Bradshaw N, Porteous M: **Integrated care pathways.** *BMJ* 1998, **316:** 133-137. | Review does not contain manuscripts concerning discharge interventions explicitly targeted to smoothen transition from hospital to home |
| Campbell RL, Banner R, Konick-McMahan J, Naylor MD: **Discharge planning and home follow-up of the elderly patient with heart failure.** *Nurs Clin North Am* 1998, **33:** 497-513. | Not a systematic review |
| Cifu DX, Stewart DG: **Factors affecting functional outcome after stroke: a critical review of rehabilitation interventions.** *Arch Phys Med Rehabil* 1999, **80:** S35-S39. | Mean methodological score <5 |
| Coleman EA: **Falling through the cracks: challenges and opportunities for improving transitional care for persons with continuous complex care needs.** *J Am Geriatr Soc* 2003, **51:** 549-555. | Not a systematic review |
| Colle F, Palese A, Brusaferro S: **[Continuity of care with written information and dedicated nurses: a literature review. Part 1].** *Assist Inferm Ric* 2004, **23:** 179-185. | Mean methodological score <5 |
| De Feo S, Opasich C: **[Educating and communicating: non-pharmacologic treatment for patients with chronic heart failure?].** *Monaldi Arch Chest Dis* 2002, **58:** 51-53. | Not a systematic review |
| Department for Evaluation in Healthcare Organisations: *Hospital Discharge Planning*. Paris: Haute Autorité de Santé; 2001. | Mean methodological score <5 |
| Dollard J, Smith J, Thompson R, Stewart S: **Broadening the reach of cardiac rehabilitation to rural and remote Australia.** *Eur J Cardiovasc Nurs* 2004, **3:** 27-42. | Mean methodological score <5 |
| Early Supported Discharge Trialists: **Services for reducing duration of hospital care for acute stroke patients.** *Cochrane Database Syst Rev* 2002. | Review does not contain manuscripts concerning discharge interventions explicitly targeted to smoothen transition from hospital to home |
| Evans RL, Connis RT, Haselkorn JK: **Evaluating rehabilitation medicine: effects on survival, function, and home care.** *Home Health Care Serv Q* 1997, **16:** 35-53. | Mean methodological score <5 |
| Fasken LL, Wipke-Tevis DD, Sagehorn KK: **Factors associated with unplanned readmissions following cardiac surgery.** *Prog Cardiovasc Nurs* 2001, **16:** 107-115. | Review does not contain manuscripts concerning discharge interventions explicitly targeted to smoothen transition from hospital to home |
| Fiorenza D, Vitacca M, Clini E: **Hospital monitoring, setting and training for home non invasive ventilation.** *Monaldi Arch Chest Dis* 2003, **59:** 119-122. | Review does not contain manuscripts concerning discharge interventions explicitly targeted to smoothen transition from hospital to home |
| Gray DP, Evans P, Sweeney K, Lings P, Seamark D, Seamark C *et al*.: **Towards a theory of continuity of care.** *J R Soc Med* 2003, **96:** 160-166. | Review does not contain manuscripts concerning discharge interventions explicitly targeted to smoothen transition from hospital to home |
| Griffiths P, Wilson-Barnett J: **The effectiveness of 'nursing beds': a review of the literature.** *J Adv Nurs* 1998, **27:** 1184-1192. | Does not concern discharge home from an acute general hospital |
| Haggerty JL, Reid RJ, Freeman GK, Starfield BH, Adair CE, McKendry R: **Continuity of care: a multidisciplinary review.** *BMJ* 2003, **327:** 1219-1221. | Does not concern discharge home from an acute general hospital |
| Harrison MB, Toman C, Logan J: **Hospital to home evidence-based education for CHF.** *Can Nurse* 1998, **94:** 36-42. | Not a systematic review |
| Hedges G, Grimmer K, Moss J, Falco J: **Performance indicators for discharge planning: a focused review of the literature.** *Aust J Adv Nurs* 1999, **16:** 20-28. | Mean methodological score <5 |
| Heseltine D: **Community outreach rehabilitation.** *Age Ageing* 2001, **30 Suppl 3:** 40-42. | Not a systematic review |
| Hoban V: **Discharge planning.** *Nurs Times* 2004, **100:** 20-22. | Not a systematic review |
| Hurst S: **Multidisciplinary discharge planning.** *Prof Nurse* 1996, **12:** 113-116. | Not a systematic review |
| Jackson MF: **Discharge planning: issues and challenges for gerontological nursing. A critique of the literature.** *J Adv Nurs* 1994, **19:** 492-502. | Not a systematic review |
| Jensen MB, Hessov I: **Nutrition and rehabilitation after discharge from the hospital: accelerating the rehabilitation with nutrition and physical training.** *Nutrition* 2000, **16:** 619-621. | Not a systematic review |
| Johnson A, Sandford J, Tyndall J: **Written and verbal information versus verbal information only for patients being discharged from acute hospital settings to home.** *Cochrane Database Syst Rev* 2004. | No studies with adults included |
| Kehlet H, Wilmore DW: **Multimodal strategies to improve surgical outcome.** *Am J Surg* 2002, **183:** 630-641. | Not a systematic review |
| Kendall MB, Ungerer G, Dorsett P: **Bridging the gap: transitional rehabilitation services for people with spinal cord injury.** *Disabil Rehabil* 2003, **25:** 1008-1015. | Not a systematic review |
| Keon WJ, Sherrard H: **Early release after cardiac surgery.** *Coron Artery Dis* 1997, **8:** 235-241. | Not a systematic review |
| Lane D, Carroll D, Lip GY: **Psychology in coronary care.** *QJM* 1999, **92:** 425-431. | Not a systematic review |
| Langhorne P: **Organisation of acute stroke care.** *Br Med Bull* 2000, **56:** 436-443. | Not a systematic review |
| Lawler K, Terregino CA: **Guidelines for evaluation and education of adult patients with mild traumatic brain injuries in an acute care hospital setting.** *J Head Trauma Rehabil* 1996, **11:** 18-28. | Not a systematic review |
| Lim WK, Lambert SF, Gray LC: **Effectiveness of case management and post-acute services in older people after hospital discharge.** *Med J Aust* 2003, **178:** 262-266. | Not a systematic review |
| Louis AA, Turner T, Gretton M, Baksh A, Cleland JG: **A systematic review of telemonitoring for the management of heart failure.** *Eur J Heart Fail* 2003, **5:** 583-590. | Review does not contain manuscripts concerning discharge interventions explicitly targeted to smoothen transition from hospital to home |
| Maramba PJ, Richards S, Myers AL, Larrabee JH: **Discharge planning process: applying a model for evidence-based practice.** *J Nurs Care Qual* 2004, **19:** 123-129. | Mean methodological score <5 |
| Marsden J: **Cataract: the role of nurses in diagnosis, surgery and aftercare.** *Nurs Times* 2004, **100:** 36-40. | Review does not contain manuscripts concerning discharge interventions explicitly targeted to smoothen transition from hospital to home |
| McAlister F, Lawson F, Teo K, rmstrong P: **A systematic review of randomized trials of disease management programs in heart failure.** *Am J Med* 2001, **110:** 378-384. | Not a systematic review |
| McArthur-Rouse F: **Critical care outreach services and early warning scoring systems: a review of the literature.** *J Adv Nurs* 2001, **36:** 696-704. | Review does not contain manuscripts concerning discharge interventions explicitly targeted to smoothen transition from hospital to home |
| McDonald K, Ledwidge M: **Heart failure management programs: can we afford to ignore the inpatient phase of care?** *J Card Fail* 2003, **9:** 258-262. | Not a systematic review |
| Mistiaen P, Duijnhouwer E, Ettema T: **The construction of a research model on post-discharge problems based on a review of the literature 1990-1995.** *Soc Work Health Care* 1999, **29:** 33-68. | Mean methodological score <5 |
| Mitchell G, Del MC, Francis D: **Does primary medical practitioner involvement with a specialist team improve patient outcomes? A systematic review.** *Br J Gen Pract* 2002, **52:** 934-939. | Does not concern discharge home from an acute general hospital |
| Moore SM: **Effects of interventions to promote recovery in coronary artery bypass surgical patients.** *J Cardiovasc Nurs* 1997, **12:** 59-70. | Mean methodological score <5 |
| Morgan D, Reed J, Palmer A: **Moving from hospital into a care home--the nurse's role in supporting older people.** *J Clin Nurs* 1997, **6:** 463-471. | Does not concern discharge home from an acute general hospital |
| Mountain G, Pighills A: **Pre-discharge home visits with older people: time to review practice.** *Health Soc Care Community* 2003, **11:** 146-154. | Not a systematic review |
| Muhlestein JB: **Post-hospitalization management of high-risk coronary patients.** *Am J Cardiol* 2000, **85:** 13B-20B. | Review does not contain manuscripts concerning discharge interventions explicitly targeted to smoothen transition from hospital to home |
| Naylor MD: **Transitional care of older adults.** *Annu Rev Nurs Res* 2002, **20:** 127-147. | Mean methodological score <5 |
| Nelson JR: **The importance of postdischarge telephone follow-up for hospitalists: a view from the trenches.** *Am J Med* 2001, **111:** 43S-44S. | Not a systematic review |
| New Zealand Guidelines Group (NZGG): *Life after stroke. New Zealand guideline for management of stroke.* Wellington: New Zealand Guidelines Group (NZGG); 2003. | Review does not contain manuscripts concerning discharge interventions explicitly targeted to smoothen transition from hospital to home |
| New Zealand Health Technology Assessment: *What is the efficacy of discharge planning protocols*. 2002. | Does not concern discharge home from an acute general hospital |
| O'Connell B, Kristjanson L, Orb A: **Models of integrated cancer care: a critique of the literature.** *Aust Health Rev* 2000, **23:** 163-178. | Mean methodological score <5 |
| O'Connor G: **Discharge planning in rehabilitation following surgery for a stoma.** *Br J Nurs* 2003, **12:** 800-807. | Review does not contain manuscripts concerning discharge interventions explicitly targeted to smoothen transition from hospital to home |
| Olsen L, Wagner L: **From vision to reality: how to actualize the vision of discharging patients from a hospital, with an increased focus on prevention.** *Int Nurs Rev* 2000, **47:** 142-156. | Review does not contain manuscripts concerning discharge interventions explicitly targeted to smoothen transition from hospital to home |
| Pande RU, Nader ND, Donias HW, D'Ancona G, Karamanoukian HL: **REVIEW: Fast-Tracking Cardiac Surgery.** *Heart Surg Forum* 2003, **6:** 244-248. | Review does not contain manuscripts concerning discharge interventions explicitly targeted to smoothen transition from hospital to home |
| Panno JM, Kolcaba K, Holder C: **Acute care for elders (ACE): a holistic model for geriatric orthopaedic nursing care.** *Orthop Nurs* 2000, **19:** 53-60. | Review does not contain manuscripts concerning discharge interventions explicitly targeted to smoothen transition from hospital to home |
| Patterson CJ, Mulley GP: **The effectiveness of predischarge home assessment visits: a systematic review.** *Clin Rehabil* 1999, **13:** 101-104. | Mean methodological score <5 |
| Payne S, Kerr C, Hawker S, Hardey M, Powell J: **The communication of information about older people between health and social care practitioners.** *Age Ageing* 2002, **31:** 107-117. | Mean methodological score <5 |
| Pulignano G, Carmenini E, Del SD, Vlasic J, Tesorio MG, Di LM *et al*.: **[Management programs for elderly patients with chronic heart failure].** *Clin Ter* 2003, **154:** 199-206. | Not a systematic review |
| Read C: **Early discharge schemes for hysterectomy patients.** *Nurs Stand* 1996, **10:** 43-45. | Not a systematic review |
| Renehan AG, Egger M, Saunders MP, O'Dwyer ST: **Impact on survival of intensive follow up after curative resection for colorectal cancer: Systematic review and meta-analysis of randomised trials.** *Br Med J* 2002, **324:** 813-816. | Review does not contain manuscripts concerning discharge interventions explicitly targeted to smoothen transition from hospital to home |
| Renholm M, Leino-Kilpi H, Suominen T: **Critical pathways. A systematic review.** *J Nursing Administration* 2002, **32:** 196-202. | Mean methodological score <5 |
| Rice-Oxley M, Turner-Stokes L: **Effectiveness of brain injury rehabilitation.** *Clin Rehabil* 1999, **13 Suppl 1:** 7-24. | Not a systematic review |
| Ringbaek TJ, Eriksen N, Vestbo J: **[Assisted home care of patients with exacerbation of COPD. Earlier discharge to treatment, monitoring and care at home led by a respiratory nurse].** *Ugeskr Laeger* 2003, **165:** 2091-2095. | Review does not contain manuscripts concerning discharge interventions explicitly targeted to smoothen transition from hospital to home |
| Rosenberg CH, Popelka GM: **Post-stroke rehabilitation. A review of the guidelines for patient management.** *Geriatrics* 2000, **55:** 75-81. | Not a systematic review |
| Schiemann D, Moers M, Blumenberg P, Schemann J: *Expertenstandard entlassungsmanagement in der Pflege*. Osnabruck: Deutschen Netzwerk fur Qualitatsentwicklung in der Pflege; 2004. | Mean methodological score <5 |
| Scottish Intercollegiate Guidelines Network: *Management of patients with stroke. Rehabilitation, prevention and management of complications and discharge planning. A National Clinical Guideline*, 64 edn. Edinburgh: SIGN; 2002. | Not a systematic review |
| Shipton S: **Risk factors associated with multiple hospital readmissions.** *Home Care Provid* 1996, **1:** 83-85. | Review does not contain manuscripts concerning discharge interventions explicitly targeted to smoothen transition from hospital to home |
| Shipton SL: **Congestive heart failure readmission.** *Home Care Provid* 1997, **2:** 171-175. | Review does not contain manuscripts concerning discharge interventions explicitly targeted to smoothen transition from hospital to home |
| Smith S: **Discharge planning: the need for effective communication.** *Nurs Stand* 1996, **10:** 39-41. | Not a systematic review |
| Stewart S, Horowitz JD: **Specialist nurse management programmes: economic benefits in the management of heart failure.** *Pharmacoeconomics* 2003, **21:** 225-240. | Not a systematic review |
| Stroke Unit Trialists Collaboration: **How do stroke units improve patient outcomes? A collaborative systematic review of the randomized trials.** *Stroke* 1997, **28:** 2139-2144. | No outcomes measured within 3 months |
| Stuck AE, Siu AL, Wieland GD, Adams J, Rubenstein LZ: **Comprehensive geriatric assessment: a meta-analysis of controlled trials.** *Lancet* 1993, **342:** 1032-1036. | Not a systematic review |
| Stuck AE: **[Multidimensional geriatric assessment in the acute hospital and ambulatory practice].** *Schweiz Med Wochenschr* 1997, **127:** 1781-1788. | Mean methodological score <5 |
| Taraborelli P, Wood F, Pithouse A, Bloor M, Parry O: *Hospital Discharge for Frail Older People: A Literature Review with Practice Case Studies*, 24 edn. The Scottish Office; 1998. | Not a systematic review |
| Tullis A, Nicol M: **A systematic review of the evidence for the value of functional assessment of older people with dementia.** *Br J Occup Ther* 1999, **62:** 554-563. | Review does not contain manuscripts concerning discharge interventions explicitly targeted to smoothen transition from hospital to home |
| van de Walle - van de Geijn B, Kruijswijk Jansen J: **De verpleegkundige als ontslagmanager : literatuuronderzoek naar problemen tijdens de ontslagfase en mogelijke oplossingen.** *Oncologica* 2003, **20:** 32-36. | Not a systematic review |
| Veterans Health Administration DoD: *VA/DoD clinical practice guideline for the management of stroke rehabilitation in the primary care setting*. Washington: Veterans Health Administration, Department of Defense.; 2003. | Review does not contain manuscripts concerning discharge interventions explicitly targeted to smoothen transition from hospital to home |
| Weir R. Rehabilitation of cerebrovascular disorder (stroke): early discharge and support. A critical appraisal of the literature. NZHTA REPORT 2[1]. 1999. | Mean methodological score <5 |
| Wielandt T, Strong J: **Compliance with prescribed adaptive equipment: A literature review.** *Br J Occup Ther* 2000, **63:** 65-75. | Review does not contain manuscripts concerning discharge interventions explicitly targeted to smoothen transition from hospital to home |
| Williams A, Botti M: **Issues concerning the on-going care of patients with comorbidities in acute care and post-discharge in Australia: a literature review.** *J Adv Nurs* 2002, **40:** 131-140. | Not a systematic review |
| Winkley A, Gostick J, Iyavoo L, Garnett C, Lonsdale J: *Ensuring the effective discharge of older patients from NHS acute hospitals*, HC 392 Session 2002-2003 edn. London: National Audit Office; 2003. | Not a systematic review |
| Zwicker D, Picariello G: **Discharge planning for the older adult.** In *Geriatric nursing protocols for best practice*. Edited by Fulmer T, Abraham I, Zwicker D. New York: Springer Publishing Company; 2003. | Not a systematic review |
