# Supplementary material for: Interventions aimed at reducing problems in adult patients discharged from hospital to home: a systematic meta-review
Source: BMC Health Serv Res. 2007 Apr 4;7:47. doi: 10.1186/1472-6963-7-47 (PMC1853085; doi:10.1186/1472-6963-7-47)
Supplement: Additional file 4 — Appendix 4: List of references of primary studies included in one of the reviews. The data show the references of the primary studies included in one of the reviews [file 1472-6963-7-47-S4.doc]

Appendix 4: List of references of primary studies included in one of the reviews

| REFERENCES INCLUDED PRIMARY STUDIES | **Cameron 2002** | **Cole 2001** | **Day 2004** | **Gwadry 2004** | **Handoll 2003** | **Hyde 2000** | **Kwan 2002** | **OST 2003** | **Parker SG 2002** | **Parker G 2000** | **Phillips 2004** | **Richards 2003** | **Shepperd 2004** | **Shepperd 2001** | **Teasel 2003** | Times cited |
| --- | --- | --- | --- | --- | --- | --- | --- | --- | --- | --- | --- | --- | --- | --- | --- | --- |
| Number of references included in reviews | 19 | 11 | 57 | 8 | 21 | 14 | 20 | 15 | 71 | 45 | 19 | 23 | 11 | 23 | 15 |  |
| Total number of references included in one or more reviews: 267  Mean 1.4; Min-max 1-5 |  |  |  |  |  |  |  |  |  |  |  |  |  |  |  |  |
|  |  |  |  |  |  |  |  |  |  |  |  |  |  |  |  |  |
| Abrami G, Stevens J. Early weight bearing after internal fixation of transcervical fracture of the femur. Preliminary report of a clinical trial. *J Bone Joint Surg.* 1964; 46(2): 204-205. |  |  |  |  | X |  |  |  |  |  |  |  |  |  |  | 1 |
| Adler MW, Waller JJ, Creese A, Thorne SC. Randomised controlled trial of early discharge for inguinal hernia and varicose veins. *J Epidemiol Community Health.* 1978; 32: 136-142. |  |  |  |  |  |  |  |  |  |  |  |  |  | X |  | 1 |
| Aitken PD, Rodgers H, French JM, et al. General medical or geriatric unit care for acute stroke? A controlled trial. *Age and Ageing.* 1993; 22: 4-5. |  |  |  |  |  |  |  |  |  | X |  |  |  |  |  | 1 |
| Alessi CA, Stuck AE, Aronow HU, et al. The process of care in preventive in-home comprehensive geriatric assessment. *J Am Geriatric Soc.* 1997; 45: 1044-1050. |  |  | X |  |  |  |  |  |  |  |  |  |  |  |  | 1 |
| Allen CM, Becker PM, McVey LJ, Saltz C, Feussner JR, Cohen HJ. A randomized, controlled clinical trial of a geriatric consultation team. Compliance with recommendations. *JAMA.* 1986; 255(19): 2617–21. |  |  |  |  |  |  |  |  | X |  |  |  |  |  |  | 1 |
| Andersen HE, Scultz-Larsen K, Kreiner S et al. Can readmission after stroke be prevented? Results of a randomized clinical study: a post discharge follow-up service for stroke survivors. *Stroke.* 2000; 31: 1038-1045. |  |  |  |  |  |  |  | X |  |  |  |  |  |  |  | 1 |
| Anderson C, Mhurchu CN, Rubenach S, et al. Home or hospital for stroke rehabilitation? Results of a randomized controlled trial. II: cost minimization analysis at 6 months. *Stroke.* 2000; 31: 1032-1037. |  |  |  |  |  |  |  |  |  |  |  |  |  |  | X | 1 |
| Anderson C, Rubenach S, Mhurchu CN et al. Home or hospital for stroke rehabilitation? Results of a randomized controlled trial. I: Health outcomes at 6 months. *Stroke.* 2000; 31: 1024-1031. |  |  |  |  |  |  |  |  |  |  |  |  |  |  | X | 1 |
| Applegate WB, Akins D, Beaver TB, et al. A randomized, controlled trial of a geriatric assessment and rehabilitation unit. *J Am Geriatric Soc.* 1988; 336: 578. |  |  |  |  |  |  |  |  |  | X |  |  |  |  |  | 1 |
| Applegate WB, Graney MJ, Miller ST, et al. Impact of a geriatric assessment unit on subsequent health care charges. *Am J Public Health.* 1991; 81: 1302-1306. |  |  |  |  |  |  |  |  |  |  |  | X |  |  |  | 1 |
| Applegate WB, Miller ST, Graney MJ, et al. A randomized, controlled trial of a geriatric assessment unit in a community rehabilitation hospital. *New Eng J Med.* 1990; 38: 1073-1081. |  |  |  |  |  |  |  |  |  |  |  | X |  |  |  | 1 |
| Applegate WB, Miller ST, Graney MJ, et al. A randomized, controlled trial of a geriatric assessment unit in a community rehabilitation hospital. *New Eng J Med.* 1990; 322: 1572-1578. |  |  | X |  |  |  |  |  |  |  |  |  |  |  |  | 1 |
| Avlund K, Jepsen E, Vass M, Lundemark H. Effects off comprehensive follow-up home visits after hospitalization on functional ability and readmissions among old patients: a randomized controlled study. *Scandinavian J Occ Ther.* 2002; 9: 17-22. |  |  | X |  |  |  |  |  |  |  |  |  |  |  |  | 1 |
| Baker CM, Miller I, Sitterding M, Hajewski CJ. Acute stroke patients: comparing outcomes with and without case management. *Nursing Case Management.* 1998; 3(5): 196-203. |  |  |  |  |  |  | X |  |  |  |  |  |  |  |  | 1 |
| Baker PA, Evans OM, Lee C. Treadmill gait retraining following fractured neck-of-femur. *Arch Phys Med Rehab.* 1991; 72(9): 649-652. |  |  |  |  | X |  |  |  |  |  |  |  |  |  |  | 1 |
| Batehup L, Griffiths P, Miller F, et al. Outcomes based evaluation of a nursing led intermediate care unit: full project report. London: King’s College London. 1998. |  |  |  |  |  |  |  |  |  | X |  |  |  |  |  | 1 |
| Bautz-Holler E, Sveen u, Rygh J et al. Early supported discharge of patients with acute stroke: a randomized controlled trial. *Disabil Rehabil.* 2002; 24: 348-355. |  |  |  |  |  |  |  |  |  |  |  |  |  |  | X | 1 |
| Beckie T. A supportive-educative telephone program: impact on knowledge and anxiety after coronary artery bypass graft surgery. *Heart Lung.* 1989; 18(1): 46–55. |  |  |  |  |  |  |  |  | X |  |  |  |  |  |  | 1 |
| Beech R, Rudd AG Tilling, K, Wolfe CDA. Economic consequences of early inpatient discharge to community-based rehabilitation for stroke in an inner London teaching hospital. *Stroke.* 1999; 30: 729–35. |  |  |  |  |  |  |  |  | X |  |  |  |  | X | X | 3 |
| Bernabei R, Landi F, Gambassi G, et al. Randomised trial of impact of model of integrated care and case management for older people living in the community. *BMJ.* 1998; 316: 1348-1351. |  |  | X |  |  |  |  |  |  |  |  |  |  |  |  | 1 |
| Blue L, Lang E, McMurray JJ, et al. Randomized controlled trial of specialist nurse intervention in heart failure. *BMJ*. 2001; 323: 715-718 |  |  |  |  |  |  |  |  |  |  | X |  |  |  |  | 1 |
| Board N, Brennan N, Caplan G. A randomised controlled trial of the costs of hospital as compared with hospital at home for acute medical patients. *Aus New Zealand J Public Health.* 2000; 24: 305-311. |  |  |  |  |  |  |  |  |  |  |  |  |  | X |  | 1 |
| Bogardus ST, Desai MM, Williams CS, et al. The effects of a targeted multicom­ponent delirium intervention on postdischarge outcomes for hospitalized older adults. *Am J Med.* 2003; 114: 383-390. |  |  | X |  |  |  |  |  |  |  |  |  |  |  |  | 1 |
| Borok GM, Reuben DB, Zendle LJ, et al. Rationale and design of a multicenter randomized trial of comprehensive geriatric assessment consultation for hospitalized patients in a HMO. *J Am Geriatric Soc.* 1994; 42: 536-544. |  |  |  |  |  |  |  |  |  |  |  | X |  |  |  | 1 |
| Boult C, Boult L, Morishita L, et al. A controlled trial of outpatient geriatric evaluation and management. *J Am Geriatric Soc.* 1994; 42: 465-470. |  |  | X |  |  |  |  |  |  |  |  |  |  |  |  | 1 |
| Boult C, Boult L, Morishita L, et al. A controlled trial of outpatient geriatric evaluation and management. *J Am Geriatric Soc.* 2001; 49: 351-359. |  |  | X |  |  |  |  |  |  |  |  |  |  |  |  | 1 |
| Bowen J, Yaste C. Effect of a stroke protocol on hospital costs of stroke patients. *Neurology.* 1994; 44: 1961-1964. |  |  |  |  |  |  | X |  |  |  |  |  |  |  |  | 1 |
| Burch S, Longbottom J, McKay M, et al. A randomized controlled trial of day hospital and day centre therapy. *Clinical Rehabilitation.* 1999; 13: 105-112. |  |  | X |  |  |  |  |  |  | X |  |  |  |  |  | 2 |
| Burns A, Park K. Proximal femoral fractures in the female patient: a controlled trial: the role of the occupational therapist and the physiotherapist. *Br J Occ Ther.* 1992; 55(10): 397-400. | X |  |  |  |  |  |  |  |  |  |  |  |  |  |  | 1 |
| Burns R, Nichols LO, Graney MJ, Cloar FT. Impact of continued geriatric outpatient management on health outcomes of older veterans. *Arch Int Med.* 1995; 155: 1313-1318 |  |  | X |  |  |  |  |  |  |  |  | X |  |  |  | 2 |
| Burns R, Nichols LO, Martindale-Adams J, Graney MJ. Interdisciplinary geriatric primary care evaluation and management: two-years outcomes. *J Am Geriatric Soc.* 2000; 48: 8-13. |  |  | X |  |  |  |  |  |  |  |  | X |  |  |  | 2 |
| Cameron ID, Lyle DM, Quine S. Accelerated rehabilitation after proximal femoral fracture: a randomized controlled trial. *Disability and Rehabilitation.* 1993; 15: 29-34. | X |  |  |  |  |  |  |  |  |  |  |  |  |  |  | 1 |
| Cameron ID, Lyle DM, Quine S. Cost effectiveness of accelerated rehabilitation after proximal femoral fracture. *J Clin Epidemiology.* 1994; 47: 1307-1313. | X |  |  |  |  |  |  |  |  |  |  |  |  |  |  | 1 |
| Caplan GA, Ward J, Brennan NJ, et al. Hospital in the home: a randomised controlled trial. *Med J Australia.* 1999; 170: 156-160. |  |  |  |  |  |  |  |  |  | X |  |  |  | X |  | 2 |
| Capomolla S, Febo O, Ceresa M, et al. Cost/utility ratio in chronic heart failure management deliverd by day hospital and usual care. *J Am Coll Cardiol.* 2002; 40: 1259-1266 |  |  |  |  |  |  |  |  |  |  | X |  |  |  |  | 1 |
| Cavalieri TA, Chopra A, Gray-Miceli D, et al. Geriatric assessment teams in nursing homes : do they work ? *J Am Osteopathic Ass.* 1993; 93: 1269-1272. |  |  | X |  |  |  |  |  |  |  |  |  |  |  |  | 1 |
| Cline CMJ, Israelsson BYA, Willenheimer RB, Broms K, Erhardt LR. Cost effective management programme for heart failure reduces hospitalisation. *Heart London.* 1998; 80(5): 442–6. |  |  |  | X |  |  |  |  | X |  | X |  |  |  |  | 3 |
| Coast J, Richards SH, Peters TJ, Gunnell DJ, Darlow M, Pounsford J. Hospital at home or acute hospital care? A cost minimisation analysis. *BMJ.* 1998; 316: 1802–6. |  |  |  |  |  |  |  |  | X |  |  |  |  | X |  | 2 |
| Cohen HJ, Feussner JR, WeinbergerM, et al. A controlled trial of inpatient and out­patient geriatric evaluation and management. *New Engl J Med.* 2002; 346: 905-912. |  |  | X |  |  |  |  |  |  |  |  |  |  |  |  | 1 |
| Corr S, Bayer A. Occupational therapy for stroke patients after hospital discharge: a randomized controlled trial. *Clinical Rehabilitation.* 1995; 9: 291-296. |  |  |  |  |  |  |  | X |  |  |  |  |  |  |  | 1 |
| Counsell SR, Holder CM, Liebenauer LL, et al. A randomized controlled trial of a community hospital ACE unit. *J Am Geriatric Soc.* 1998; 46: 9, A24. |  |  |  |  |  |  |  |  |  | X |  |  |  |  |  | 1 |
| Counsell SR, Holder CM, Liebenauer LL, et al. Effects of a multicomponent intervention on functional outcomes and process of care in hospitalized older patients: a randomized controlled trial of Acute Care for Elders (ACE) in a community hospital. *J Am Geriatric Soc.* 2000; 48: 1572-1581. |  |  | X |  |  |  |  |  |  |  |  |  |  |  |  | 1 |
| Crawley WD. Case management: improving outcomes of care for ischaemic stroke patients. *MEDSURG Nursing.* 1996; 5(4): 239-244. |  |  |  |  |  |  | X |  |  |  |  |  |  |  |  | 1 |
| Davies L, Wilkinson M, Bonner S et al. Hospital at home versus hospital care in patients with exacerbations of chronic pulmonary disease: prospective randomised controlled trial. *BMJ.* 2000; 321: 1265-1268. |  |  |  |  |  |  |  |  |  |  |  |  |  | X |  | 1 |
| Day GA, Swanson C, Yelland C, et al. Surgical outcomes of a randomized prospective trial involving patients with a proximal femoral fracture. *Australian & New Zealand J Surgery.* 2001; 71(1): 11-14. | X |  |  |  |  |  |  |  |  |  |  |  |  |  |  | 1 |
| Day GA, Yelland C, Swanson CE, et al. Early rehabilitation in patients with hip fractures [abstract]. *J Bone Joint Surgery. British Volume.* 1997; 79(suppl IV): 410. | X |  |  |  |  |  |  |  |  |  |  |  |  |  |  | 1 |
| Donald IP, Baldwin RN, Bannerjee M. Gloucester hospital-at-home: a randomized controlled trial. *Age Ageing* 1995; 24(5): 434–9. |  | X |  |  |  | X |  |  | X | X |  |  |  | X |  | 5 |
| Drummond AER, Walker Mf. A randomized controlled trial of leisure rehabilitation after stroke. *Clin Rehab.* 1995; 9: 283-290. |  |  |  |  |  |  |  | X |  |  |  |  |  |  |  | 1 |
| Drummond AER, Walker MF. Generalisation of the effects of leisure rehabilitation for stroke patients. *Br j Occ Ther.* 1996; 59(7): 330-334. |  |  |  |  |  |  |  | X |  |  |  |  |  |  |  | 1 |
| Duncan P, Richards L, Wallace D et al. A randomized controlled pilot study of a home-based exercise program for individuals with mild and moderate stroke. *Stroke.* 1998; 29: 2055-2060. |  |  |  |  |  |  |  | X |  |  |  |  |  |  | X | 2 |
| Dunn RB, Guy PM, Hardman CS, Lewis PA, Vetter NJ. Can a house call by a public health nurse improve the quality of the discharge process for geriatric patients? *Clin Perform Qual Health Care.* 1995; 3: 151–5. |  |  |  |  |  | X |  |  | X |  |  |  |  |  |  | 2 |
| Dunn RB, Lewis PA, Vetter NJ, Guy PM, Hardman CS, Jones RW. Health visitor intervention to reduce days of unplanned hospital re-admission in patients recently discharged from geriatric wards: The results of a randomised controlled study. *Arch Gerontol Geriatr.* 1994; 18(1): 15–23. |  |  |  |  |  | X |  |  | X |  |  |  |  |  |  | 2 |
| Eagle DJ, Guyatt GH, Patterson C, et al. Effectiveness of a geriatric day hospital. *CMAJ.* 1991; 144: 699-704. |  |  | X |  |  |  |  |  |  | X |  |  |  |  |  | 2 |
| Ekman I, Andersson B, Ehnfors M et al. Feasibility of a nurse-monitored, outpatient-care programme for elderly patients with moderate-to-severe chronic heart failure. *Eur Heart J.* 1998; 19: 1254-1260. |  |  |  | X |  |  |  |  |  |  |  |  |  |  |  | 1 |
| Eng C, Pedulla J, Eleazer GP et al. Program of All-inclusive Care for the Elderly (PACE): an innovative model of integrated geriatric care and financing. *J Am Geriatric Soc.* 1997; 45: 223-232. |  |  | X |  |  |  |  |  |  |  |  |  |  |  |  | 1 |
| Engelhardt JB, Toseland RW, O’Donnell JC, Richie JT, et al. The effectiveness and efficiency of outpatient geriatric evaluation and management. *J Am Geriatric Soc.* 1996; 44: 847-856. |  |  | X |  |  |  |  |  |  |  |  |  |  |  |  | 1 |
| Epstein AM, Hall JA, Fretwell M, et al. Consultative geriatric assessment for ambulatory patients: a randomized trial in a health maintenance organization. *JAMA.* 1990; 263: 538-544. |  |  | X |  |  |  |  |  |  |  |  |  |  |  |  | 1 |
| Evans RL, Hendricks RD. Evaluating hospital discharge planning: a randomized clinical trial. *Med Care.* 1993; 31(4): 358–70. |  |  |  |  |  |  |  |  | X |  |  |  | X |  |  | 2 |
| Fabacher D, Josephson K, Pietruszka K, et al. An in-home preventive assessment program for independent older adults: a randomized controlled trial. *J Am Geriatric Soc.* 1994; 42: 630-638. |  |  | X |  |  |  |  |  |  |  |  |  |  |  |  | 1 |
| Falconer JA, Roth EJ, Sutin JA et al. The critical path method in stroke rehabilitation: lessons from an experiment in cost containment and outcome improvement. *Quality Review Bulletin.* 1993; 19: 8-16. |  |  |  |  |  |  | X |  |  |  |  |  |  |  |  | 1 |
| Fishman LM, Emro MA. Active use of serial functional assessment improves outcome and shortens acute geriatric hospitalization. *Top Geriatr Rehabil.* 1994; 9(3): 16–29. |  |  |  |  |  |  |  |  | X |  |  |  |  |  |  | 1 |
| Fitzgerald JF, Smith DM, Martin DK, Freedman JA, Katz BP. A case manager intervention to reduce readmissions. *Arch Intern Med.* 1994; 154(15): 1721–9. |  |  |  |  |  |  |  |  | X |  |  |  |  |  |  | 1 |
| Fordham R, Thompson R, Holmes J, Hodkinson C. A cost-benefit study of geriatric-orthopedic management of patients with fractured neck of femur. Discussion paper 14. York: Centre for health Economics. University of York, 1986. | X |  |  |  |  |  |  |  |  |  |  |  |  |  |  | 1 |
| Fretwell MD, Raymond PM, McGarvey ST, Owens N, Traines M, Silliman RA, *et al*. The senior care study. A controlled trial of a consultative/unit-based geriatric assessment program in acute care. *J Am Geriatr Soc.* 1990; 38(10): 1073–81. |  |  |  |  |  |  |  |  | X | X |  | X |  |  |  | 3 |
| Galvard H, Samuelsson SM. Orthopaedic or geriatric rehabilitation of hip fracture patients: a prospective, randomized, clinically controlled study in Malmo, Sweden. *Aging (Milano).* 1995; 7(1): 11-16. | X |  |  |  |  |  |  |  |  | X |  |  |  |  |  | 2 |
| Gilbertson L, Langhorne P, Walker A et al. Domiciliary occupational therapy for patients with stroke discharged from hospital: randomised controlled trial. *BMJ.* 2000; 320: 603-606. |  |  |  |  |  |  |  | X |  |  |  |  |  |  |  | 1 |
| Gilchrist WJ, Newman RJ, Hamblen DL, Williams BO. Prospective randomised study of an orthopaedic geriatric inpatient service. *BMJ.* 1988; 297: 1116-1118. | X |  |  |  |  |  |  |  |  | X |  |  |  |  |  | 2 |
| Gilliss CL, Gortner SR, Hauck WW, Shinn JA, Sparacino PA, Tompkins C. A randomized clinical trial of nursing care for recovery from cardiac surgery. *Heart Lung.* 1993; 22(2): 125–33. |  |  |  |  |  |  |  |  | X |  |  |  |  |  |  | 1 |
| Gladman JR, Lincoln NB, Barer DH. A randomised controlled trial of domiciliary and hospital-based rehabilitation for stroke patients after discharge from hospital. *J Neurol Neurosurg Psychiatry.* 1993; 56(9): 960–6. |  |  |  |  |  |  |  |  | X | X |  |  |  |  |  | 2 |
| Gladman JR, Lincoln NB. Follow-up of a controlled trial of domiciliary stroke rehabilitation (DOMINO Study). *Age Ageing.* 1994; 23(1): 9–13. |  |  |  |  |  |  |  |  | X |  |  |  |  |  |  | 1 |
| Goldberg G, Segal ME, Berk SN et al. Stroke transition after inpatient rehabilitation. *Top stroke Rehabil.* 1997; 4(1): 64-79. |  |  |  |  |  |  |  | X |  |  |  |  |  |  |  | 1 |
| Graham J. Early or delayed weight-bearing after internal fixation of transcervical fracture of the femur: a clinical trial. *J Bone Joint Surg.* 1968; 50(3): 562-569. |  |  |  |  | X |  |  |  |  |  |  |  |  |  |  | 1 |
| Grande GE, Todd CJ, Barclay Si, Farquhar MC. A randomised controlled trial of a hospital at home service for the terminally ill. *Palliative Medicine.* 2000; 14: 375-385. |  |  |  |  |  |  |  |  |  |  |  |  |  | X |  | 1 |
| Grande GE, Todd CJ, Barclay SI, Farquhar MC. Does hospital at home for palliative care facilitate death at home? Randomised controlled trial. *BMJ.* 1999. 319; 1472-75. |  |  |  |  |  |  |  |  |  |  |  |  |  | X |  | 1 |
| Griffiths P. Evaluation of nurse led inpatient care. *Nursing Times.* 1995; 91: 34-37. |  |  |  |  |  |  |  |  |  | X |  |  |  |  |  | 1 |
| Gwadry-Sridhar F, Arnold JMO, Guyatt G et al. A randomized controlled trial of a multi-disciplinary inpatient team intervention in patients with heart failure. Paper presented at Canadian Cardiovascular Congres, Alberta. 2004. |  |  |  | X |  |  |  |  |  |  |  |  |  |  |  | 1 |
| Hamrin EKF, Lindmark B. The effect of systematic care planning after acute stroke in general hospital medical wards. *J Adv Nurs.* 1990; 15: 1146-1153. |  |  |  |  |  |  | X |  |  |  |  |  |  |  |  | 1 |
| Hankey GJ, Deleo D, Stewart-Wynne EG. Acute hospital care for stroke patients: a randomised trial. *Cerebrovascular Disease.* 1995; 5: 228. |  |  |  |  |  |  |  |  |  | X |  |  |  |  |  | 1 |
| Hansen FR, Poulsen H, Sorensen KH. A model of regular geriatric follow-up by home visits to selected patients discharged from a geriatric ward: a randomized controlled trial. *Aging (Milano).* 1995; 7(3): 202–6. |  |  | X |  |  | X |  |  | X |  |  |  |  |  |  | 3 |
| Hansen FR, Spedtsberg K, Schroll M. Geriatric follow-up by home visits after dis­charge from hospital: a randomized controlled trial. *Age Ageing.* 1992; 21(6): 445–50. |  |  |  |  |  | X |  |  | X |  |  |  |  |  |  | 2 |
| Harris RD, Henschke PJ, Popplewell PY, et al. A randomised study of outcomes in a defined group of acutely ill elderly patients managed in a geriatric assessment unit or a general medical unit. *Aust N Z J Med* 1991; 21(2): 230–4. |  |  |  |  |  |  |  |  | X | X |  |  |  |  |  | 2 |
| Harrison MB, Browne Gb, Roberts J, et al. Quality of life of individuals with heart failure: a randomized trial of the effectiveness of two models of hospital-to-home transition. *Med Care* 2002; 40: 271-282. |  |  |  |  |  |  |  |  |  |  | X |  |  |  |  | 1 |
| Hauer K, Pfisterer M, Schuler M, et al. Two years later: a prospective long-term follow-up of a training intervention in geriatric patients with a history of severe falls. *Arch Phys Med Rehab.* 2003; 84(10): 1426-1432. |  |  |  |  | X |  |  |  |  |  |  |  |  |  |  | 1 |
| Hauer K, Rost B, Rutschle K, et al. Exercise training for rehabilitation and secondary prevention of falls in geriatric patients with a history of injurious falls. *J Am Geriatr Soc.* 2001; 49(1): 10-20. |  |  |  |  | X |  |  |  |  |  |  |  |  |  |  | 1 |
| Hauer K, Specht N, Schuler M, et al. Intensive physical training in geriatric patients after severe falls and hip surgery. *Age Ageing.* 2002; 31(1): 49-57. |  |  |  |  | X |  |  |  |  |  |  |  |  |  |  | 1 |
| Hedrick SC, Rothman Ml, Chapko M, et al. Summary and discussion of methods and results of the adult day health care evaluation study. *Medical Care.* 1993; 31(suppl 9): SS94-103. |  |  |  |  |  |  |  |  |  | X |  |  |  |  |  | 1 |
| Hendriksen C, Stromgard E, Sorensen K. [Current cooperation concerning admission to and discharge from geriatric hospitals]. *Nordisk Medicin.* 1990; 105: 58-60. |  |  |  |  |  |  |  |  |  |  |  |  | X |  |  | 1 |
| Hogan DB, Fox RA, Badley BW, Mann OE. Effect of a geriatric consultation service on management of patients in an acute care hospital. *CMAJ.* 1987; 136(7): 713–17. |  |  |  |  |  |  |  |  | X |  |  | X |  |  |  | 2 |
| Hogan DB, MacDonald FA, Betts J, et al. A randomized controlled trial of a community-based consultation service to prevent falls. *CMAJ.* 2001; 165: 537-543. |  |  | X |  |  |  |  |  |  |  |  |  |  |  |  | 1 |
| Hughes SL, Cummings J, Weaver F, et al. A randomized trial of the cost effectiveness of VA hospital-based home care for the terminally ill. *Health Serv Res.* 1992; 26: 801-817. |  |  |  |  |  |  |  |  |  |  |  |  |  | X |  | 1 |
| Hui E, Lum CM, Woo J, Or KH, Kay RL. Outcomes of elderly stroke patients. Day hospital versus conventional medical management. *Stroke* 1995; 26(9): 1616–19. |  |  |  |  |  |  |  | X | X | X |  |  |  |  | X | 4 |
| Hui E, Woo J. Telehealth for older patients: the Hong Kong experience: *J Telemedicine and Telecare.* 2002; 8(suppl 3): 39-41. |  |  | X |  |  |  |  |  |  |  |  |  |  |  |  | 1 |
| Huusko TM, Karppi P, Avikainen V, et al. Randomised clinically controlled trial of intensive geriatric rehabilitation in patients with hip fracture: subgroup analysis of patients with dementia. *BMJ.* 2000; 321: 1107-1111. | X |  |  |  |  |  |  |  |  |  |  |  |  |  |  | 1 |
| Huusko TM, Karppi P, Avikainen V, et al.. Intensive geriatric rehabilitation of hip fracture patients. *Acta Orthopaedica Scandinavica.* 2002; 73(4): 425-431. | X |  |  |  |  |  |  |  |  |  |  |  |  |  |  | 1 |
| Indredavik B, Bakke F, Slordahl SA et al. Treatment in a combined acute and rehabilitation stroke unit. *Stroke.* 2000; 30: 917-923. |  |  |  |  |  |  |  |  |  |  |  |  |  | X |  | 1 |
| Indredavik B, Bakke F, Solberg R, et al. Benefit of a stroke unit: a randomised controlled trial. *Stroke.* 1991; 22: 1026-1031. |  |  |  |  |  |  |  |  |  | X |  |  |  |  |  | 1 |
| Indredavik B, Fjaertoft H, Ekeberg G et al. Benefit of an extended stroke unit service with early supported discharge: a randomized controlled trial. *Stroke.* 2000; 31: 2989-2994. |  |  |  |  |  |  |  |  |  |  |  |  |  |  | X | 1 |
| Inouye SK, Bogardus ST, Baker DI et al. The hospital Elder Life Program: a model of care to prevent cognitive and functional decline in older hospitalized patients. *J Am Geriatric Soc.* 2000; 48: 1697-1706. |  |  | X |  |  |  |  |  |  |  |  |  |  |  |  | 1 |
| Inouye SK, Bogardus ST, Charpentier PA et al. A multicomponent intervention to prevent delirium in hospitalized older patients. *New England J Med.* 1999; 340: 669-676. |  |  | X |  |  |  |  |  |  |  |  |  |  |  |  | 1 |
| Inouye SK. Prevention of delirium in hospitalized older patients: risk factors and targeted intervention strategies. *Annals of Medicine.* 2000; 32: 257-263. |  |  | X |  |  |  |  |  |  |  |  |  |  |  |  | 1 |
| Jaarsma T, Halfens R, Huijer Abu-Saad H, et al., Effects of education and support on self-care and resource utilization in patients with heart failure, *Eur Heart J* 1999(20): 673-82 |  |  |  | X |  |  |  |  |  |  | X |  |  |  |  | 2 |
| Jones J, Wilson A, Parker H et al. Economic evaluation of hospital at home versus hospital care: cost minimisation analysis of data from randomised controlled trial. *BMJ.* 1999; 319: 1547-1550. |  |  |  |  |  |  |  |  |  |  |  |  |  | X |  | 1 |
| Jongbloed L, Morgan D. An investigation of involvement in leisure activities after stroke. *Am J Occ Ther.* 1991; 45(5): 420-427. |  |  |  |  |  |  |  | X |  |  |  |  |  |  |  | 1 |
| Juby LC, Lincoln NB, Berman P, et al. The effect of a stroke rehabilitation unit on functional and psychological outcome: a randomised controlled trial. *Cerebrovascular Disease.* 1996; 6: 106-110. |  |  |  |  |  |  |  |  |  | X |  |  |  |  |  | 1 |
| Kalra L, Dale P, Crome P. Improving stroke rehabilitation: a controlled study. *Stroke.* 1993; 24: 1462-1467. |  |  |  |  |  |  |  |  |  | X |  |  |  |  |  | 1 |
| Kalra L, Eade J. Role of stroke rehabilitation units in managing severe disability after stroke. *Stroke.* 1995; 26: 2031-2034. |  |  |  |  |  |  |  |  |  | X |  |  |  |  |  | 1 |
| Karppi P, Tilvis R. Effectiveness of a Finnish geriatric inpatient assessment. Two-year follow up of a randomized clinical trial on community-dwelling patients. *Scan J Primary Health Care.* 1995; 13: 93-98. |  |  | X |  |  |  |  |  |  |  |  |  |  |  |  | 1 |
| Karppi P. Effects of a geriatric inpatient unit on elderly home care patients: a controlled trial. *Aging (Milano).* 1995; 7: 207-211. |  |  | X |  |  |  |  |  |  |  |  |  |  |  |  | 1 |
| Karumo I. Recovery and rehabilitation of elderly subjects with femoral neck fractures. *Annales Chirurgiae et Gynaecologiae.* 1977; 66(3): 170-176. |  |  |  |  | X |  |  |  |  |  |  |  |  |  |  | 1 |
| Karumo I. A randomized trial of postoperative physiotherapy after meniscectomy and fractures of the femoral neck and shaft [thesis]. Helsinki: University of Helsinki. 1978. |  |  |  |  | X |  |  |  |  |  |  |  |  |  |  | 1 |
| Kaste M, Palomaki H, Sarna S. Where and how should elderly stroke patients be treated? A randomized trial. *Stroke.* 1995; 26: 249-253. |  |  |  |  |  |  |  |  |  | X |  |  |  |  |  | 1 |
| Kay G, MacTavish M, Moffatt C, Lau G. Development and evaluation of a geriatric assessment unit in a community hospital. *Perspectives.* 1992; 16: 2-9. |  |  |  |  |  |  |  |  |  | X |  |  |  |  |  | 1 |
| Kennedy L, Neidlinger S, Scroggins K. Effective comprehensive discharge planning for hospitalized elderly. *Gerontologist* 1987; 27(5): 577–80. |  |  |  |  |  |  |  |  | X |  |  | X | X |  |  | 3 |
| Kennie DC, Reid J, Richardson IR, et al. Effectiveness of geriatric rehabilitative care after fractures of the proximal femur in elderly women: a randomised clinical trial. *BMJ.* 1988; 297: 1083-1086. | X |  |  |  |  |  |  |  |  | X |  |  |  |  |  | 2 |
| Kravitz RL, Reuben DB, Davis JW, et al. Geriatric home assessment after hospital discharge. *J Am Geriatric Soc.* 1994; 42: 1229-1234. |  |  | X |  |  |  |  |  |  |  |  |  |  |  |  | 1 |
| Krumholz HM, Amatruda J, Smith Gl, et al. Randomized trial of an education and support intervention to prevent readmission of patients with heart failure. *J Am Coll Cardiol.* 2002; 39: 83-89 |  |  |  |  |  |  |  |  |  |  | X |  |  |  |  | 1 |
| Kwan J. Integrated care pathways for acute stroke: an evaluation of their effects using multiple approaches [thesis]. University of Edinburgh, 2002. |  |  |  |  |  |  | X |  |  |  |  |  |  |  |  | 1 |
| Kwan J, Hand P, Dennis M, Sandercock P. What are the effects of introducing a care pathway in an acute stroke unit? Results of before-and-after study of 351 patients. *Cerebrovascular Diseases.* 2002; 13(supll 3): 7. |  |  |  |  |  |  | X |  |  |  |  |  |  |  |  | 1 |
| Kwan J, Hand P, Dennis M, Sandercock P. Effects of introducing an integrated care pathway in an acute stroke unit. *Age Ageing.* 2004; 33: 362-367. |  |  |  |  |  |  | X |  |  |  |  |  |  |  |  | 1 |
| Lamb SE, Grimley Evans J, Morse RE, Trundle H. A randomised placebo controlled trial and double blind study of neuromuscular stimulation to improve mobility in the first three months after surgical fixation for proximal femoral fracture [abstract]. *J Bone Joint Surg.* 1998; 80(suppl II): 172 |  |  |  |  | X |  |  |  |  |  |  |  |  |  |  | 1 |
| Lamb SE, Oldham JA, Morse RE, Evans JG. Neuromuscular stimulation of the quadriceps muscle after hip fracture: a randomized controlled trial. *Arch Phys Med Rehab.* 2002; 83(8): 1087-1092. |  |  |  |  | X |  |  |  |  |  |  |  |  |  |  | 1 |
| Landefeld CS, Palmer RM, Kresevic DM, Fortinsky RH, Kowal J. A randomized trial of care in a hospital medical unit especially designed to improve the functional outcomes of acutely ill older patients. *N Engl J Med* 1995; 332(20): 1338–44. |  |  |  |  |  |  |  |  | X | X |  |  |  |  |  | 2 |
| Landi F, Gambassi G, Pola R, et al. Impact of integrated home care services on hospital use. *J Am Geriatric Soc.* 1999; 47: 1430-1434. |  |  | X |  |  |  |  |  |  |  |  |  |  |  |  | 1 |
| Laramee AS, Levinsky SK, Sargent J, et al. Case management in a heterogeneous congestive heart failure population: a randomized controlled trial. *Arch Intern Med.* 2003; 163: 809-817. |  |  |  |  |  |  |  |  |  |  | X |  |  |  |  | 1 |
| Lauridsen UB, de la Cour BB, Gottschalk L, Svensson BH. Intensive physical therapy after hip fracture. *Danish Med Bull.* 2002; 49(1): 70-72. |  |  |  |  | X |  |  |  |  |  |  |  |  |  |  | 1 |
| Lauridsen UB, de la Cour BB, Gottschalk L, Svensson BH. [Intensive physical therapy after trochanteric femoral fracture: a randomized clinical trial]. *Ugeskrift for Laeger.* 2002; 164(8): 1040-1044. |  |  |  |  | X |  |  |  |  |  |  |  |  |  |  | 1 |
| Lim W, Lambert S, Gray L. Effectiveness of case management and post-acute services in older people after hospital discharge. *Med J Australia.* 2003; 178: 262-266. |  |  | X |  |  |  |  |  |  |  |  |  |  |  |  | 1 |
| Lipton HL, Bird JA. The impact of clinical pharmacists’ consultations on geriatric patients’ compliance and medical care use: a randomized controlled trial. *Gerontologist* 1994; 34(3): 307–15. |  |  |  |  |  |  |  |  | X |  |  |  |  |  |  | 1 |
| Logan PA, Ahern J, Gladman JR, Lincoln NB. A randomized controlled trial of enhanced Social Service occupational therapy for stroke patients. *Clin Rehab* 1997; 11(2): 107–13. |  |  |  |  |  |  |  | X | X |  |  |  |  |  |  | 2 |
| Lowe CJ, Raynor DK, Courtney EA, Purvis J, Teale C. Effects of self medication programme on knowledge of drugs and compliance with treatment in elderly patients. *BMJ* 1995; 310(6989): 1229–31. |  |  |  |  |  |  |  |  | X |  |  |  |  |  |  | 1 |
| Malone M, Hill A, Smith G. Three-month follow up of patients discharged from a geriatric day hospital. *Age and Ageing.* 2002; 31: 471-475. |  |  | X |  |  |  |  |  |  |  |  |  |  |  |  | 1 |
| Martin BJ. Randomised, controlled trial of additional quadriceps strength training in patients rehabilitating after a proximal femoral fracture. The National Research Register Issue 2, 2001. Oxford: Update Software. |  |  |  |  | X |  |  |  |  |  |  |  |  |  |  | 1 |
| Martin F, Oyewole A, Moloney A. A randomized controlled trial of a high support hospital discharge team for elderly people. *Age Ageing* 1994; 23(3): 228–34. |  | X |  |  |  | X |  |  | X | X |  |  |  | X |  | 5 |
| Mayo NE, Wood-Dauphinee S, Cote r et al. There’s no place like home: an evaluation of early supported discharge for stroke. *Stroke.* 2000; 31: 1016-1023. |  |  |  |  |  |  |  |  |  |  |  |  |  |  | X | 1 |
| McDonald K, Ledwige M, Cahill J, et al. Heart failure management: multidisciplinary care has intrinsic benefit above the optimization of medical care. *J Card Fail* 2002, 8: 142-148 |  |  |  |  |  |  |  |  |  |  | X |  |  |  |  | 1 |
| McInnes E, Mira M, Atkin N, Kennedy P, Cullen J. Can GP input into discharge planning result in better outcomes for the frail aged: results from a randomised controlled trial. *Fam Pract* 1999; 16: 289–93. |  |  |  |  |  |  |  |  | X |  |  | X |  |  |  | 2 |
| McNamee P, Christensen J, Soutter J, Rodgers H, Craig N, Pearson P, *et al*. Cost analysis of early supported hospital discharge for stroke. *Age Ageing* 1998; 27(3): 345–51. |  |  |  |  |  |  |  |  | X |  |  |  |  |  |  | 1 |
| McVey LJ, Becker PM, Saltz CC, Feussner JR, Cohen HJ. Effect of a geriatric consultation team on functional status of elderly hospitalized patients. A randomized, controlled clinical trial. *Ann Intern Med* 1989; 110(1): 79–84. |  |  |  |  |  |  |  |  | X |  |  |  |  |  |  | 1 |
| Melin AL, Bygren LO. Efficacy of the rehabilitation of elderly primary health care patients after short-stay hospital treatment. *Med Care* 1992; 30(11): 1004–15. |  | X |  |  |  | X |  |  | X | X |  |  |  |  |  | 4 |
| Melin AL, Bygren LO. Perceived functional health of frail elderly in a primary home care programme and correlation of self-perception with objective measurements. *Scand J Soc Med* 1993; 21(4): 256–63. |  |  |  |  |  | X |  |  | X |  |  |  |  |  |  | 2 |
| Melin AL, Hakansson S, Bygren LO. The costeffectiveness of rehabilitation in the home: a study of Swedish elderly. *Am J Public Health* 1993; 83(3): 356–62. |  |  | X |  |  | X |  |  | X |  |  |  |  |  |  | 3 |
| Melin AL, Wieland D, Harker JO, Bygren LO. Health outcomes of post-hospital in-home team care: secondary analysis of a Swedish trial. *J Am Geriatr Soc* 1995; 43(3): 301–7. |  |  | X |  |  | X |  |  | X |  |  |  |  |  |  | 3 |
| Mitchell SL, Stott DJ, Martin BJ, Grant SJ. Randomised controlled trial of quadriceps training after proximal femoral fracture. *Clin Rehab.* 2001; 15(3): 282-290. |  |  |  |  | X |  |  |  |  |  |  |  |  |  |  | 1 |
| Mitchell SL, Stott DJ, Martin BJ, Grant SJ. Randomised controlled trial of quadriceps training after proximal femoral fracture [abstract]. *Age Ageing.* 1999; 28(supll 2): 81. |  |  |  |  | X |  |  |  |  |  |  |  |  |  |  | 1 |
| Moher D, Weinberg A, Hanlon R, Runnalls K, Effects of a medical team coordinator on length of hospital stay. *Can Med Assoc J.* 1992; 146(4): 511-515. |  |  |  |  |  |  |  |  |  |  |  |  | X |  |  | 1 |
| Mosimaneotsile B, Braun K, Tokishi C. Stroke patients outcomes: does an integrated delivery model of care make a difference? *Physical and Occupational therapy in Geriatrics.* 2000; 17(2): 67-82. |  |  |  |  |  |  | X |  |  |  |  |  |  |  |  | 1 |
| Mor V, Granger CV, Sherwood CC. Discharged rehabilitation patients: impact of follow-up surveillance by a friendly visitor. *Arch Phys Med Rehabil* 1983; 64(8): 346. |  | X |  |  |  |  |  |  | X |  |  |  |  |  |  | 2 |
| Naglie G, Goldlist B, Etchells E, et al. A randomized trial of interdisciplinary care on an orthopaedic-geriatric unit for elderly hip fracture patients [abstract]. *Gerontologist.* 1999; 39(special issue): 420. | X |  |  |  |  |  |  |  |  |  |  |  |  |  |  | 1 |
| Naglie G, Tansey C, Kirkland JL, et al. Interdisciplinary inpatient care for elderly people with hip fracture: a randomized controlled trial. *Can Med Ass J.* 2002; 167(1): 25-32. | X |  |  |  |  |  |  |  |  |  |  |  |  |  |  | 1 |
| Naughton BJ, Moran MB, Feinglass J, Falconer J, Williams ME. Reducing hospital costs for the geriatric patient admitted from the emergency department: a randomized trial. *J Am Geriatr Soc* 1994; 42(10): 1045–9. |  |  |  |  |  |  |  |  | X |  |  |  | X |  |  | 2 |
| Naylor M, Brooten D, Jones R, Lavizzo Mourey R, Mezey M, *et al*. Comprehensive discharge planning for the hospitalized elderly. A randomized clinical trial. *Ann Intern Med* 1994; 120(12): 999–1006. |  |  |  |  |  |  |  |  | X |  | X | X | X |  |  | 4 |
| Naylor MD, Brooten D, Campbell R, Jacobsen BS, Mezey MD, Pauly MV, *et al*. Comprehensive discharge planning and home follow-up of hospitalized elders: a randomized clinical trial. *JAMA* 1999; 281(7): 613–20. |  | X |  |  |  |  |  |  | X |  | X | X |  |  |  | 4 |
| Naylor MD. Comprehensive discharge planning for hospitalized elderly: a pilot study. *Nurs Res* 1990; 39(3): 156–61. |  |  |  |  |  |  |  |  | X |  |  |  |  |  |  | 1 |
| Nazareth I, Burton A, Shulman S, Smith P, Haines A. A pharmacy discharge plan for hospitalized elderly patients: a randomized controlled trial. *Age and Ageing.* 2001; 30: 33-40. |  |  |  |  |  |  |  |  |  |  |  |  | X |  |  | 1 |
| Neidlinger SH, Scroggins K, Kennedy LM. Cost evaluation of discharge planning for hospitalized elderly: the efficacy of a clinical nurse specialist. *Nurs Econ* 1987; 5(5): 225–30. |  |  |  |  |  |  |  |  | X |  |  |  |  |  |  | 1 |
| Newman RJ, Gilchrist WJ, Hamblen DL, et al. A prospective randomised study of an orthopaedic-geriatric inpatient service [abstract]. *J Bone Joint Surgery.* 1989; 79: 871. | X |  |  |  |  |  |  |  |  |  |  |  |  |  |  | 1 |
| Nielsen M, Blenker M, Bloom M. Older persons after hospitalisation. A controlled study of home aid services. *Am J Public Health* 1972; 62: 1094–101. |  | X |  |  |  | X |  |  | X |  |  |  |  |  |  | 3 |
| Nikolaus T, Specht-Leible N, Bach M, et al. A randomized trial of comprehensive geriatric assessment and home intervention in the care of hospitalized patients. *Age and Ageing.* 1999; 28: 543-550 |  | X | X |  |  |  |  |  |  |  |  | X |  |  |  | 3 |
| Nikolaus T, Specht-Leible N, Bach M, et al. Effectiveness of hospital-based geriatric evaluation and management and home intervention team (GEM-HIT). Rationale and design of a 5-year randomized trial. *Zeitschrift Geron Geriatrie.* 1995; 28: 47-53. |  |  |  |  |  |  |  |  |  | X |  | X |  |  |  | 2 |
| Odderson IR, McKenna BS. A model for management of patients with stroke during the acute phase: outcome and economic implications. *Stroke.* 1993; 17(2): 1823-1827. |  |  |  |  |  |  | X |  |  |  |  |  |  |  |  | 1 |
| Oddone EZ, Weinberger M, Giobbie-Hurder A, et al. Enhanced access to primary care for patients with congestive heart failure. *Eff Clin Pract* 1999; 2: 201-209 |  |  |  |  |  |  |  |  |  |  | X |  |  |  |  | 1 |
| Oster P, Hauer K, Specht N, et al. [Muscle strength and coordination training for prevention of falls in elderly patients]. *Zeitschrift Geront Geriatrie.* 1997; 30: 289-92. |  |  |  |  | X |  |  |  |  |  |  |  |  |  |  | 1 |
| Parfrey PS, Gardner E, Vavasour H, Harnelt JD, McManamon C, McDonald J. The feasibility and efficacy of early discharge planning initiated by the admitting department in two acute care hospitals. *Clin Invest Med* 1999; 17(2): 88–96. |  |  |  |  |  |  |  |  | X |  |  |  | X |  |  | 2 |
| Parker CJ, Gladman JRF, Drummond AER et al. A multicentre randomized controlled trial of leisure therapy and conventional occupational therapy after stroke. *Clin Rehab.* 2001; 15: 42-52. |  |  |  |  |  |  |  | X |  |  |  |  |  |  |  | 1 |
| Pasquarello MA. Measuring the impact of an acute stroke program on patient outcomes. *J Neuroscience Nurs.* 1990; 22(2): 76-82. |  |  |  |  |  |  | X |  |  |  |  |  |  |  |  | 1 |
| Pearson A, Punton S, Durant I. Nursing beds: an evaluation of the effects of therapeutic nursing. Harrow: Scutari Press. 1992. |  |  |  |  |  |  |  |  |  | X |  |  |  |  |  | 1 |
| Pearson A, Punton S, Durant I. The feasibility and effectiveness of nursing beds. *Nursing Times.* 1988; 84: 48-50. |  |  |  |  |  |  |  |  |  | X |  |  |  |  |  | 1 |
| Pereles L, Romonko L, Murzyn T, Hogan D, Silvius J, Stokes E, *et al*. Evaluation of a selfmedication program. *J Am Geriatr Soc* 1996; 44(2): 161–5. |  |  |  |  |  |  |  |  | X |  |  |  |  |  |  | 1 |
| Phillips CY. Postdischarge follow-up care: effect on patient outcomes. *J Nurs Care Qual* 1993; 7(4): 64–72. |  |  |  |  |  |  |  |  | X |  |  |  |  |  |  | 1 |
| Pitkala K. The effectiveness of day hospital care on home care patients. *J Am Geriatric Soc.* 1998; 46: 1086-1090. |  |  | X |  |  |  |  |  |  |  |  |  |  |  |  | 1 |
| Quine S, Helby L, Cameron I, Lyle D. Carer burden after proximal femoral fracture. *Disability and Rehabilitation.* 1994; 16: 191-197. | X |  |  |  |  |  |  |  |  |  |  |  |  |  |  | 1 |
| Rainville EC, Impact of pharmacist interventions on hospital readmissions for heart failure. *Am J Health Syst Pharm* 1999; 56: 442-446 |  |  |  |  |  |  |  |  |  |  | X |  |  |  |  | 1 |
| Rawl SM, Easton KL, Kwiatkowski S, Zemen D, Burczyk B. Effectiveness of a nurse-managed follow-up program for rehabilitation patients after discharge. *Rehabil Nurs* 1922; 23(4): 204–9. |  |  |  |  |  |  |  |  | X |  |  |  |  |  |  | 1 |
| Reid J, Kennie DC. Geriatric rehabilitative care after fractures of the proximal femur: one year follow-up of a randomised clinical trial. *BMJ.* 1989; 299: 25-26. | X |  |  |  |  |  |  |  |  |  |  |  |  |  |  | 1 |
| Reuben DB, Borok GM, et al. A randomized trial of comprehensive geriatric assess­ment in the care of hospitalized patients. *New Eng J Med.* 1995; 332: 1345-1350 |  |  | X |  |  |  |  |  |  |  |  | X |  |  |  | 2 |
| Ricauda NA, Pla LF, Marinello R, et al. Feasibility of an acute stroke home care service for elderly patients. *Arch Gerontology Geriatrics.* 1998; Suppl 6: 17-22. |  |  |  |  |  |  |  |  |  | X |  |  |  | X | X | 3 |
| Rich MW, Beckham V, Wittenberg C, Leven CE, Freedland KE, Carney RM. Repetitive hospital admissions for congestive heart failure in the elderly. *Am J Geriatr Cardiol.* 1996; 5(3): 32–6. |  |  |  |  |  |  |  |  | X |  |  |  |  |  |  | 1 |
| Rich MW, Beckham V, Wittenberg C, Leven CL, Freedland KE, Carney RM. A multidisciplinary intervention to prevent the readmission of elderly patients with congestive heart failure. *N Engl J Med.* 1995; 333(18): 1190–5. |  | X |  | X |  |  |  |  | X |  | X |  |  |  |  | 4 |
| Rich MW, Gray DB, Beckham V, Wittenberg C, Luther P. Effect of a multidisciplinary intervention on medication compliance in elderly patients with congestive heart failure. *Am J Med.* 1996; 101(3): 270–6. |  |  |  |  |  |  |  |  | X |  |  |  |  |  |  | 1 |
| Rich MW, Vinson JM, Sperry JC, Shah AS, Spinner LR, Chung MK, *et al*. Prevention of readmission in elderly patients with congestive heart failure: results of a prospective, randomized pilot study. *J Gen Intern Med.* 1993; 8(11): 585–90. |  |  |  | X |  |  |  |  | X |  | X |  |  |  |  | 3 |
| Richards SH, Coast J, Gunnell DJ, Peters TJ, Pounsford J, Darlow M. Randomised controlled trial comparing effectiveness and acceptability of an early discharge, hospital at home scheme with acute hospital care. *BMJ.* 1998; 316(7147): 1796–801. |  |  |  |  |  |  |  |  | X | X |  |  |  | X |  | 3 |
| Riegel B, Carlson B, Kopp Z, et al. Effect of standardized nurse case-management telephone intervention on resource use in patients with chronic heart failure. *Arch Intern Med.* 2002; 162: 705-712 |  |  |  |  |  |  |  |  |  |  | X |  |  |  |  | 1 |
| Ritchie C, Wieland D, Tully C, et al. Coordination and advocacy for rural elders (CARE): a model of rural case management with veterans. *Gerontologist.* 2002; 42: 399-405. |  |  | X |  |  |  |  |  |  |  |  |  |  |  |  | 1 |
| Rizzo JA, Bogardus ST, Leo-Summers L, et al. Multicomponent targeted intervention to prevent delirium in hospitalized older patients: what is the economic value? *Medical Care.* 2001; 39: 740-752. |  |  | X |  |  |  |  |  |  |  |  |  |  |  |  | 1 |
| Rockwood K, Stadnyk K, Carver D, et al. A clinimetric evaluation of specialized geriatric care for rural dwelling, frail older people. *J Am Geriatric Soc.* 2000; 48: 1080-1085. |  |  | X |  |  |  |  |  |  |  |  |  |  |  |  | 1 |
| Rodgers H, Soutter J, Kaiser W, Pearson P, Dobson R, Skilbeck C, *et al*. Early supported hospital discharge following acute stroke: pilot study results. *Clin Rehabil* 1997; 11(4): 280–7. |  |  |  |  |  |  |  |  | X | X |  |  |  | X | X | 4 |
| Rogers H. Development of an early supported hospital discharge policy following acute stroke – an evaluation. Final report. University of Newcastle-upon-Tyne, 1997. |  |  |  |  |  |  |  |  | X |  |  |  |  |  |  | 1 |
| Ronning OM, Guldvog B. Outcome of a subacute stroke rehabilitation: a randomized controlled trial. *Stroke.* 1998; 29: 779-784. |  |  |  |  |  |  |  |  |  | X |  |  |  |  |  | 1 |
| Ronning OM, Guldvog B. Stroke units versus general medical wards. I: twelve and eighteen-month survival. *Stroke.* 1998; 29: 58-62. |  |  |  |  |  |  |  |  |  | X |  |  |  |  |  | 1 |
| Ronning OM, Guldvog B. Stroke units versus general medical wards. II: neurological deficits and activities of daily living; a quasi-randomized controlled trial. *Stroke.* 1998; 29: 586-590. |  |  |  |  |  |  |  |  |  | X |  |  |  |  |  | 1 |
| Ross G, Johnson D, Kobernick M. Evaluation of a critical pathway for stroke. *J Am Osteopathic Ass.* 1997; 97(5): 269-276. |  |  |  |  |  |  | X |  |  |  |  |  |  |  |  | 1 |
| Rubenstein LZ, Josephson KR, Harker JO etal. The Sepulveda GEU study revisited: long term outcomes, use of services, and costs. *Aging (Milano).* 1995; 7: 212-217. |  |  | X |  |  |  |  |  |  |  |  | X |  |  |  | 2 |
| Rubenstein LZ, Josephson KR, Wieland GD, English PA, Sayre JA, Kane RL. Effectiveness of geriatric evaluation unit. A randomized clinical trial. *N Engl J Med* 1984; 311(26): 1664–70. |  |  | X |  |  |  |  |  | X | X |  | X |  |  |  | 4 |
| Rubenstein LZ, Wieland GD, Josephson KR, et al. Improved survival for frail elderly inpatients on a geriatric evaluation unit (GEU): who benefits? *J Clin Epidemiology.* 1988; 41: 441-449. |  |  | X |  |  |  |  |  |  |  |  |  |  |  |  | 1 |
| Rubin CD, Francis J. Evaluation and management of geriatric outpatients. *Annals Int Med.* 1994; 120 (6 suppl 2): 40. |  |  |  |  |  |  |  |  |  |  |  | X |  |  |  | 1 |
| Rubin CD, Sizemore MT, Loftis PA, Adams Huet B, Anderson RJ. The effect of geriatric evaluation and management on Medicare reimbursement in a large public hospital: a randomized clinical trial. *J Am Geriatr Soc.* 1992; 40(10): 989–95. |  |  |  |  |  |  |  |  | X |  |  | X |  |  |  | 2 |
| Rubin CD, Sizemore MT, Loftis PA, de Mola NL. A randomized trial of outpatient geriatric evaluation and management in a large public hospital. *J Am Geriatr Soc.* 1993; 41: 1023-1028. |  |  | X |  |  |  |  |  |  |  |  | X |  |  |  | 1 |
| Ruckley CV, Cuthbertson C, Fenwick N et al. day care operations for hernia or varicose veins: a controlled trial. *Br J Surg.* 1978; 65: 456-459. |  |  |  |  |  |  |  |  |  |  |  |  |  | X |  | 1 |
| Rudd AG, Wolfe CDA, Tilling K, Beech R. Randomised controlled trial to evaluate early discharge scheme for patients with stroke. *BMJ* 1997; 315(7115): 1039–44. |  |  |  |  |  |  |  |  | X | X |  |  |  | X | X | 4 |
| Salgado R, Ehrlich F, Banks C, et al. A mobile rehabilitation team program to assist patients in nursing homes rehabilitate and return to their homes. *Archives of Gerontology & Geriatrics.* 1995; 20: 255-261. |  |  | X |  |  |  |  |  |  |  |  |  |  |  |  | 1 |
| Saltvedt I, Mo ES, Fayers P et al. Reduced mortality in treating acutely sick, frail older patients in a geriatric evaluation and management unit: a prospective randomized trial. *J Am Geriatric Soc* 2002; 50: 792-798. |  |  | X |  |  |  |  |  |  |  |  |  |  |  |  | 1 |
| Saltz CC, McVey LJ, Becker PM, Feussner JR, Cohen HJ. Impact of a geriatric consultation team on discharge placement and repeat hospitalization. *Gerontologist* 1988; 28(3): 344–50. |  |  |  |  |  |  |  |  | X |  |  |  |  |  |  | 1 |
| Sanchez ferrin P, Manas Magana M, et al. [Geriatric assessment of elderly patients with proximal fracture of the femur]. *Revista Espanola de Geriatria y Gerontologia.* 1999; 34(2): 65-71. | X |  |  |  |  |  |  |  |  |  |  |  |  |  |  | 1 |
| Schull DE, Tosch P. Wood M. Clinical nurse specialist as collaborative care managers. *Nursing Management.* 1992; 23: 30-33. |  |  |  |  |  |  | X |  |  |  |  |  |  |  |  | 1 |
| Serxner S, Miyaji M, Jeffords J. Congestive heart failure disease management study: a patient education intervention. *Congest Heart Fail.* 1998; 4: 23-28 |  |  |  | X |  |  |  |  |  |  | X |  |  |  |  | 2 |
| Shaw H., Mackie CA, Sharkie I. Evaluation of effect of pharmacy discharge planning on medication problems experienced by discharged acute admission mental health problems. *The Int J Pharm Practice.* 2000; 8: 144-153. |  |  |  |  |  |  |  |  |  |  |  |  | X |  |  | 1 |
| Shepperd S, Harwood D, Gray A, et al. Randomised controlled trial comparing hospital at home care with inpatient hospital care: II. Cost minimisation analysis. *BMJ.* 1998; 316: 1791-1796. |  |  |  |  |  |  |  |  |  |  |  |  |  | X |  | 1 |
| Shepperd S, Harwood D, Jenkinson C, et al. Randomised controlled trial comparing hospital at home care with inpatient hospital care: I. Three month follow up of health outcomes. *BMJ.* 1998; 316: 1786-1791. |  |  |  |  |  |  |  |  |  | X |  |  |  | X |  | 2 |
| Sherrington C, Lord SR. Home exercise to improve strength and walking velocity after hip fracture: a randomised controlled trial. *Arch Phys Med Rehab.* 1997; 78(2): 208-212. |  |  |  |  | X |  |  |  |  |  |  |  |  |  |  | 1 |
| Sherrington C, Lord SR, Herbert RD. A randomised trial of weight-bearing versus non weight-bearing exercise for improving physical ability in inpatients after hip fracture. *Australian J Physiotherapy.* 2003; 49(1): 15-22. |  |  |  |  | X |  |  |  |  |  |  |  |  |  |  | 1 |
| Sherrington C, Lord SR, Herbert RD. The effects of exercise on physical ability following fall-related hip fracture: two randomised controlled trials. Proceedings Australian Physiotherapy Association, 7^th^ International Physiotherapy Congress, Sydney. 2002. |  |  |  |  | X |  |  |  |  |  |  |  |  |  |  | 1 |
| Sherrington C, Lord SR, Herbert RD. A randomized controlled trial of weight-bearing versus non weight-bearing exercise for improving physical ability in inpatients after usual care for hip fracture. *Arch Phys Med Rehab.* 2004; 85(5): 710-716. |  |  |  |  | X |  |  |  |  |  |  |  |  |  |  | 1 |
| Sherrington C, Lord SR, Herbert RD. A randomised controlled trial of weight-bearing versus non weight-bearing exercise for improving physical ability in inpatients after hip fracture and completion of usual care. XVIth conference of the International Society for Postural and Gait Research. Sydney, 2003. |  |  |  |  | X |  |  |  |  |  |  |  |  |  |  | 1 |
| Silliman RA, McGarvey ST, Raymond PM, Fretwell MD. The senior care study. Does inpatient interdisciplinary geriatric assessment help the family caregivers of acutely ill older patients?. *J Am Geriatric Soc.* 1990; 38: 461-466. |  |  |  |  |  |  |  |  |  |  |  | X |  |  |  | 1 |
| Silverman M, Musa D, Martin DC, et al. Evaluation of outpatient geriatric assessment: a randomized multi-site trial. *J Am Geriatric Soc.* 1995; 43: 733-740. |  |  | X |  |  |  |  |  |  |  |  |  |  |  |  | 1 |
| Siu AL, Kravitz RL, Keeler E, Hemmerling K, Kington R, Davis JW, *et al*. Postdischarge geriatric assessment of hospitalized frail elderly patients. *Arch Intern Med.* 1996; 156(1): 76–81. |  | X | X |  |  |  |  |  | X |  |  | X |  |  |  | 4 |
| Siu AL, Morishita L, Blaustein J. Comprehensive geriatric assessment in a day hospital. *J Am Geriatric Soc.* 1994; 42: 1094-1099. |  |  | X |  |  |  |  |  |  |  |  |  |  |  |  | 1 |
| Slaets JPJ, Kauffmann RH, Duivenvoorden HJ, Pelemans W, Schudel WJ. A randomized trial of geriatric liaison intervention in elderly medical inpatients. *Psychosom Med.* 1997; 59(6): 585–91. |  |  |  |  |  |  |  |  | X | X |  |  |  |  |  | 2 |
| Smith DM, Weinberger M, Katz BP, Moore PS. Postdischarge care and readmissions. *Med Care.* 1988; 26(7): 699–708. |  |  |  |  |  |  |  |  | X |  |  |  |  |  |  | 1 |
| Smith Ds, Goldenberg E, Ashburn A et al. Remedial therapy after stroke: a randomised controlled trial. *BMJ.* 1981; 282: 517-520. |  |  |  |  |  |  |  | X |  |  |  |  |  |  |  | 1 |
| Smith N. Effectiveness of geriatric rehabilitative care [letter]. *BMJ.* 1988; 297: 1609. | X |  |  |  |  |  |  |  |  |  |  |  |  |  |  | 1 |
| Stewart S, Marley JE, Horowitz, JD. Effects of a multidisciplinary, home-based intervention on planned readmissions and survival among patients with congestive heart failure: a randomised controlled study. *Lancet.* 1999; 354: 1077-83. |  |  |  |  |  |  |  |  |  |  | X |  |  |  |  | 1 |
| Stewart S, Pearson S, Horowitz, JD. Effects of a home-based intervention among patients with congestive heart failure discharged from acute hospital care. *Arch Intern Med.* 1998; 158(10): 1067–72. |  |  |  | X |  |  |  |  | X |  | X |  |  |  |  | 3 |
| Stewart S, Pearson S, Luke CG, Horowitz JD. Effects of home-based intervention on unplanned readmissions and out-of-hospital deaths. *J Am Geriatr Soc.* 1998; 46(2): 174–80. |  | X |  |  |  |  |  |  | X |  |  |  |  |  |  | 2 |
| Stewart S, Vandenbroek AJ, Pearson S, Horowitz JD. Prolonged beneficial effects of a home-based intervention on unplanned readmissions and mortality among patients with congestive heart failure. *Arch Intern Med.* 1999; 159(3): 257–61. |  |  |  |  |  |  |  |  | X |  |  |  |  |  |  | 1 |
| Strijbos JH, Postma DS, van Altena R, et al. A comparison between an outpatient hospital-based pulmonary rehabilitation program and a home-care rehabilitation program in patients with COPD: a follow up of 18 months. *Chest.* 1996; 109; 366-372. |  |  |  |  |  |  |  |  |  | X |  |  |  |  |  | 1 |
| Stuck AE, Aronow HU, Steiner A, et al. A trial of annual in-home comprehensive geriatric assessments for elderly people living in the community. *New Eng J Med.* 1995; 333: 1184-1189. |  |  | X |  |  |  |  |  |  |  |  |  |  |  |  | 1 |
| Stuck AE, Minder CE, Peter-Wuset I, et al. A randomised trial of in-home visits for disability prevention in community-dwelling older people at low and high risk for nursing home admission. *Arch Int Med.* 2000; 160: 977-986. |  |  | X |  |  |  |  |  |  |  |  |  |  |  |  | 1 |
| Styrborn K. Early discharge planning for elderly patients in acute hospitals: an intervention study. *Scan J Soc Med.* 1995; 23: 273-285. |  |  | X |  |  |  |  |  |  |  |  |  |  |  |  | 1 |
| Sulch D, Evans A, Melbourn A, Kalra L. Does an integrated care pathway improve process of care in stroke rehabilitation? A randomized controlled trial. *Age Ageing.* 2002; 31: 175-179. |  |  |  |  |  |  | X |  |  |  |  |  |  |  |  | 1 |
| Sulch D, Melbourn A, Perez I, Kalra L. Integrated care pathways and quality of life on a stroke rehabilitation unit. *Stroke.* 2002; 33: 1600-1604. |  |  |  |  |  |  | X |  |  |  |  |  |  |  |  | 1 |
| Sulch D, Perez I, Melbourn A, Kalra L. Evaluation of an integrated care pathway for stroke unit rehabilitation. *Age Ageing.* 1999; 29(suppl 1): 57. |  |  |  |  |  |  | X |  |  |  |  |  |  |  |  | 1 |
| Sulch D, Perez I, Melbourn A, Kalra L. Randomized controlled trial of integrated (managed) care pathways for stroke rehabilitation. *Stroke.* 2000; 31(8): 1929-1934. |  |  |  |  |  |  | X |  |  |  |  |  | X |  |  | 2 |
| Swanson CE, Day GA, Yelland E, et al. The management of elderly patients with femoral fractures : a randomised controlled trial of early intervention versus standard care. *Med J Australia.* 1998; 169: 515-518. | X |  |  |  |  |  |  |  |  |  |  |  |  |  |  | 1 |
| Teng J, Mayo NE, Latimer E et al. Costs and caregiver consequences of early supported discharge for stroke patients. *Stroke.* 2003; 34: 528-536. |  |  |  |  |  |  |  |  |  |  |  |  |  |  | X | 1 |
| Thomas DR, Brahan R, Haywood BP. Inpatient community-based geriatric assessment reduces subsequent mortality. *J Am Geriatr Soc.* 1993; 41(2): 101–4. |  |  |  |  |  |  |  |  | X |  |  | X |  |  |  | 2 |
| Tinetti ME, Baker D, Gallo WT et al. Evaluation of restorative care versus usual care for older adults receiving an acute episode of home care. *JAMA.* 2002; 287: 2098-2105. |  |  | X |  |  |  |  |  |  |  |  |  |  |  |  | 1 |
| Toseland RW, O’Donnell JC, Engelhardt JB, et al. Outpatient geriatric evaluation and management: results of a randomized trial. *Medical Care.* 1996; 34: 624-640. |  |  | X |  |  |  |  |  |  |  |  |  |  |  |  | 1 |
| Townsend J, Dyer S, Cooper J et al. Emergency hospital admissions and readmissions of patients aged over 75 years and the effects of a community-based discharge scheme. *Health Trends.* 1992; 24: 136-139. |  |  |  |  |  | X |  |  |  |  |  |  |  |  |  | 1 |
| Townsend J, Piper M, Frank AO, Dyer S, North WR, Meade TW. Reduction in hospital readmission stay of elderly patients by a community based hospital discharge scheme: a randomised controlled trial. *BMJ.* 1988; 297(6647): 544–7. |  | X |  |  |  | X |  |  | X |  |  |  |  |  |  | 3 |
| Trentini M, Semeraro S, Rossi E, et al. A multicenter randomized trial of comprehensive geriatric assessment and management: experimental design, baseline data and six-month preliminary results. *Aging (Milano).* 1995; 7: 224-233. |  |  |  |  |  |  |  |  |  | X |  | X |  |  |  | 2 |
| Tucker MA, Davison JG, Ogle SJ. Day hospital rehabilitation : effectiveness and cost in the elderly, a randomized controlled trial. *BMJ.* 1984; 289: 1209-1212. |  |  | X |  |  |  |  |  |  |  |  |  |  |  |  | 1 |
| von Koch L, Widen Holmqvist L, Kostulas V,et al. A randomized controlled trial of rehabilitation at home after stroke in southwest Stockholm. *Scand J Rehab Med.* 2000; 32: 80-86. |  |  |  |  |  |  |  |  |  |  |  |  |  | X | X | 2 |
| von Koch L, Pedro-Cuesta J, Kostulas V et al. Randomized controlled trial of rehabilitation at home after stroke: one-year follow-up of patient outcome, resource use and costs. *Cerebrovascular Disease.* 2001; 12: 131-138. |  |  |  |  |  |  |  |  |  |  |  |  |  |  | X | 1 |
| von Sternberg T, Hepburn K, Cibuzar P et al. Post-hospital sub-acute care: an example of a managed care model. *J Am Geriatric Soc.* 1997; 45: 87-91. |  |  | X |  |  |  |  |  |  |  |  |  |  |  |  | 1 |
| Walker MF, Drummond AER. Evaluation of dressing practice for stroke patients after discharge from hospital: a crossover design study. *Clin Rehab.* 1996; 10: 23-31. |  |  |  |  |  |  |  | X |  |  |  |  |  |  |  | 1 |
| Walker MF, Gladman JRF, Lincoln NB et al. Occupational therapy for stroke patients not admitted to hospital: a randomised controlled trial. *Lancet.* 1999; 354: 278-280. |  |  |  |  |  |  |  | X |  |  |  |  |  |  |  | 1 |
| Wee AS, Cooper WB, Chatham RK, et al. The development of a stroke clinical pathway: an experience in a medium-sized community hospital. *J Mississippi State Med Ass.* 2000; 41(7): 648-653. |  |  |  |  |  |  | X |  |  |  |  |  |  |  |  | 1 |
| Weinberger M, Oddone EZ, Henderson WG. Does increased access to primary care reduce hospital readmissions? Veterans Affairs Cooperative Study Group on Primary Care and Hospital Readmission. *N Engl J Med.* 1996; 334(22): 1441–7. |  |  |  |  |  |  |  |  | X |  | X |  | X |  |  | 3 |
| White SJ, Powers JS, Knight JR, Harrell D, Varnell L, Vaughn C, *et al.* Effectiveness of an inpatient geriatric service in a university hospital. *J Tenn Med Assoc.* 1994; 87(10): 425–8. |  |  |  |  |  |  |  |  | X |  |  |  |  |  |  | 1 |
| Widen Holmqvist L, von Koch L, Kostulas V, Holm M, Widsell G, Tegler H, *et al*. A randomized controlled trial of rehabilitation at home after stroke in southwest Stockholm. *Stroke.* 1998; 29(3): 591–7. |  |  |  |  |  |  |  |  | X | X |  |  |  | X | X | 4 |
| Widjaja LS, Chan BP, Chen H, et al. Variance analysis applied to a stroke pathway: how this can improve efficiency of healthcare delivery. *Annals Academy Medicine Singapore.* 2002; 31(4): 425-430. |  |  |  |  |  |  | X |  |  |  |  |  |  |  |  | 1 |
| Wieland D, Lamb VL, Sutton SR et al. Hospitalization in the Program of All-inclusive Care for the Elderly (PACE): rates, concomitants, and predictors. *J Am Geriatric Soc.* 2000; 48: 1373-1380. |  |  | X |  |  |  |  |  |  |  |  |  |  |  |  | 1 |
| Wilkinson G, Parcell M, MacDonald A. Finalist: ACHS Quality Improvement Award: Cerebrovascular accident clinical pathway. *J Qual Clin Pract.* 2000; 20: 109-112. |  |  |  |  |  |  | X |  |  |  |  |  |  |  |  | 1 |
| Williams EI, Greenwell J, Groom LM. The care of people over 75 years old after discharge from hospital: an evaluation of timetabled visiting by health visitor assistants. *J Public Health Med.* 1992; 14(2): 138–44. |  |  |  |  |  | X |  |  | X |  |  |  |  |  |  | 2 |
| Williams H, Blue B, Langlois PF. Do follow-up home visits by military nurses of chronically ill medical patients reduce readmissions? *Milit Med.* 1994; 159(2): 141–4. |  |  |  |  |  |  |  |  | X |  |  |  |  |  |  | 1 |
| Williams ME, Williams TF, Zimmer JG et al. How does the team approach to outpatient geriatric evaluation compare with traditional care: a report of a randomized controlled trial. *J Am Geriatric Soc.* 1987; 35: 1071-1078. |  |  | X |  |  |  |  |  |  |  |  |  |  |  |  | 1 |
| Williford SL, Johnson DF. Impact of pharmacist counseling on medication knowledge and compliance. *Milit Med.* 1995; 160(11): 561–4. |  |  |  |  |  |  |  |  | X |  |  |  |  |  |  | 1 |
| Wilson A, Parker H, Wynn A et al. Randomised controlled trial of effectiveness of Leicester hospital at home scheme compared with hospital care. *BMJ.* 1999; 319: 1542-1546. |  |  |  |  |  |  |  |  |  |  |  |  |  | X |  | 1 |
| Wilson A, Parker H, Wynn A, et al. Management of acute conditions in hospital or hospital at home: a randomised controlled trial. 1998. University of Leicester. |  |  |  |  |  |  |  |  |  | X |  |  |  |  |  | 1 |
| Winograd, CH, Gerety MB, Lai NA. A negative trial of inpatient geriatric consultation: lessons learned and recommendations for future research. *Arch Intern Med.* 1993; 1: 2017–23. |  |  |  |  |  |  |  |  | X |  |  |  |  |  |  | 1 |
| Wolfe CDA, Tilling K, Rudd AG. The effectiveness of community-based rehabili­ta­tion for stroke patients who remain at home: a pilot randomized trial. *Clin Rehab.* 2000; 14: 563-569. |  |  |  |  |  |  |  | X |  |  |  |  |  |  |  | 1 |
| Wong J, Wong S, Nolde T, Yabsley RH. Effects of an experimental program on post-hospital adjustment of early discharged patients. *Int J Nurs Stud* 1990; 27(1): 7–20. |  |  |  |  |  |  |  |  | X |  |  |  |  |  |  | 1 |
| Yeo G, Ingram L, Skurnick J, Crapo L. Effects of a geriatric clinic on functional health and well-being of elders. *J Gerontology.* 1987; 42: 252-258. |  |  | X |  |  |  |  |  |  |  |  |  |  |  |  | 1 |
| Young JB, Forster A. The Bradford community stroke trial: eight week results. *Clin Rehab.* 1991; 5: 283-289. |  |  |  |  |  |  |  |  |  | X |  |  |  |  |  | 1 |
